# A hybrid color emotional experience approach: Integrating the pleasure-arousal-dominance model with fuzzy grey relational analysis

**DOI:** 10.1371/journal.pone.0341895

**Published:** 2026-02-02

**Authors:** Tianyu Wu, Tianlu Zhu, Yiqian Zhao, Cengjuan Wu

**Affiliations:** 1 School of Art, An Hui University, Hefei, China; 2 School of Design Art and Media, Nanjing University of Science and Technology, Nanjing, China; 3 School of Design, ShangHai Jiao Tong University, Shanghai, China; G H Raisoni College of Engineering and Management, Pune, INDIA

## Abstract

Color schemes are a crucial component of modern product design and user experience, closely linked to users’ emotional needs. However, emotional experiences with product colors are inherently complex and abstract. Accurately capturing these emotional tendencies and translating them into effective color schemes has long been a challenge in emotional design. This study proposes an emotional experience-based approach to product color matching, grounded in Kansei Engineering (KE). To establish a robust closed-loop between design and evaluation, both forward and reverse KE models were developed, accompanied by comprehensive evaluation methods ranging from color factor analysis to the optimization of final schemes. Given the vagueness and complexity of the experiential data obtained from 216 questionnaires, the Pleasure-Arousal-Dominance (PAD) model was integrated with fuzzy Grey Relational Analysis (GRA) to extract nuanced and meaningful insights. Using the color design of a household hair dryer as a case study, the feasibility of the framework was demonstrated. Comparative results showed that, the proposed PAD–fuzzy GRA approach produces more stable and discriminative evaluation outcomes for intermediate schemes. Moreover, the resulting rankings exhibited a higher degree of consistency with independent eye-tracking measurements, indicating a closer alignment with users’ actual visual attention and emotional perception. The proposed methodology effectively captures users’ emotional responses to specific color schemes without relying on overly complex calculations or experimental conditions. It aligns with subjective visual preferences and identifies color combinations that evoke positive emotions. By incorporating practical design psychology methods, this hybrid design and evaluation framework offers an intuitive and applicable reference for emotional experience, extending beyond conventional color matching.

## Introduction

Color serves as a crucial medium in human-product interaction, linking products, designers, and users. In product design, color schemes extend across the entire product lifecycle. They convey both functional information and brand concepts, and they strongly influence emotional experiences [1]. Color can shape a user’s psychological response from the first encounter, and these effects may persist unconsciously [2]. It plays an irreplaceable role in shaping emotional engagement with products. Beyond practical utility, the quality and personalization of color design have become key objectives [3]. Furthermore, with the establishment of “Brand Days” in countries like the U.S. and China, brand concepts are gaining significant attention in consumer markets. Consequently, color design’s role in brand creation and communication is increasingly prominent, aiding in product differentiation and brand promotion [4].

Over the past fifty years, product color design has placed greater emphasis on the emotional impact of design schemes and their evaluation. With the evolution of modern aesthetic preferences, users’ emotional needs for product colors have become more diverse and personalized [5]. This growing variability creates uncertainty that challenges traditional design methods. It calls for more flexible and adaptive approaches [6]. Guided by Kansei Engineering (KE), researchers combined traditional methods such as Likert-scale evaluation and semantic differential (SD) analysis with advanced technologies like Grey System Theory (GST), neural networks (NNs), and genetic algorithms (GAs). These approaches aim to identify color design factors that align with users’ emotional needs and to gradually optimize product color schemes.

To assess how product colors influence users’ moods, Ding et al. [7] analyzed electroencephalogram (EEG) data, event-related potentials (ERPs), and affective evaluation scales. The study used images of colored electric hand drills. They found that color systems had a stronger impact than hue relationships. Warm colors and distinct hues evoked proactive moods. In contrast, neutral colors, similar hues, and gray tones produced ambiguous moods and slower reaction times. To improve emotion-driven color design and better capture user preferences, Zhang et al. combined GST with SD [8] and Chaos Theory with SD [9] in two distinct studies. Both approaches were demonstrated through modeling-sedan case studies. The first approach identified trends in sedan colors as macro factors and found correlations between hue, saturation, brightness, and “natural/stylish” feelings as micro factors. The second approach, using the predictability of nonlinear chaotic patterns, extracted “stylish” and “natural” as core descriptors for sedan brand images. Likewise, using Likert-scale and SD methods, Guo et al. [10] determined five key perceptual features—order, excitement, temperature, color harmony, and users’ emotional preference. They developed tricolor schemes for baby carriages and electric cars with NNs and a GA. These schemes optimized both color harmony and emotional preference, underscoring the role of color-area ratio in emotional experiences. Yeh et al. [11] and Deng et al. [12] examined sports shoes and cultural products, respectively. Their GA-based models achieved similar results, integrating user preferences to improve design efficiency, predict consumer preferences, and balance subjective and objective aesthetics. In advertising, Zhu et al. [13] examined how color and design complexity affect advertisement effectiveness using Pleasure-Arousal-Dominance (PAD) model, providing insights into matching graphics with color design to better evoke consumer emotions. It is evident that current research on product color emotional experience increasingly integrates multidisciplinary theories and methods, becoming fairly comprehensive and diverse. The focus is shifting from cognitive and perceptual color preferences to enhancing satisfaction with emotional experiences [2,7,10]. Physiological measurements and quantitative analysis, supported by big data and computing technologies, have become essential for collecting, designing, and evaluating emotional data.

While these approaches enhance the reliability of design schemes, they also face challenges related to experimental conditions and algorithmic complexity. Drawing from practical design psychology and current research, this study proposes a hybrid design method combining the PAD model with fuzzy Grey Relational Analysis (GRA). The PAD model identifies color forms that evoke positive emotions in users, while the fuzzy GRA method offers a multidimensional evaluation and optimization of design schemes. Specifically, we developed a hybrid Kansei Engineering System (HKES) framework, comprising both forward and backward KE systems, for the color design of a household hair dryer. In the forward KE system (FKES) stage, 216 participants conducted PAD scaling measurements and assessed their emotional experiences with the color-matching schemes. In the subsequent backward KE system (BKES) stage, these schemes were evaluated and optimized using fuzzy GRA. This approach aims to provide a clear theoretical reference and a practical pathway for designing product color experiences that better meet users’ emotional needs.

In light of the above review, this study investigates the following three key research questions (RQs): RQ1: how can users’ emotional responses to product colors be captured with accuracy while relying on methods that remain accessible, time-efficient, and do not require complex experimental setups or advanced equipment? Existing physiological measures are often costly or context-dependent, and there is a need for practical alternatives that maintain reliability. RQ2: how can the outcomes of emotional measurement be effectively linked to the evaluation of design schemes, thereby creating a closed-loop between “design” and “evaluation”? Bridging this gap is critical to ensure that emotional tendencies identified at the measurement stage can meaningfully inform the optimization of product color schemes. RQ3: when faced with multi-dimensional and often ambiguous emotional data, how can designers reasonably filter, prioritize, and optimize among competing objectives so that the final design schemes are both emotionally resonant and aesthetically appealing to users?

This article is structured as follows: The Literature Review section outlines the theoretical basis, reviewing Kansei Engineering, the PAD emotional model, and fuzzy grey relational analysis to establish the study’s conceptual foundation. The Methods and Framework section describes the methodology and details each step of the proposal, highlighting the integration of forward and backward Kansei systems within a hybrid framework. The Case Research and Results section presents a detailed case study using a household hair dryer, demonstrating how the proposed method is applied to real product color design and validating its feasibility. The Discussion section examins the research results, offers insights into both theoretical and practical implications, and outlines directions for future work to broaden the scope of emotional design research. The Conclusions and Limitations section summarizes the key findings and offers suggestions for future research directions.

## Literature review

### Hybrid Kansei engineering

KE was founded by M. Nagamachi in Japan in the early 1970s. It is widely used in product color emotional design due to its accessibility and practicality [14,15]. The term “kansei” is Japanese for “feeling and image”, encompassing a compound of human psychological processes influenced by societal development, fashion trends, and lifestyle concepts [14,16,17]. Via qualitative reasoning and quantitative analysis, KE practically captures users’ psychological feelings and needs regarding products, enhancing experiences and services by integrating these insights into product design.

Among the six updated KE types—category classification, KE system (KES), HKES, KE modelling, virtual KE, and collaborative KE designing [16,18,19]—HKES was noteworthy as a particularly powerful design tool. HKES comprises FKES and BKES. Specifically, FKES translates users’ potential needs into specific design elements, aligning designs more closely with user desires; while BKES evaluates and optimizes based on users’ emotional needs [18,20]. By incorporating multidisciplinary theories and technologies in design element collection and scheme evaluation, HKES facilitates the creation of product designs that align with market demands.

To address real-world multi-objective optimization, Yang [21] integrated a multi-objective genetic algorithm (MOGA) into FKES to generate product alternatives and support vector regression (SVR) for predicting emotional responses. This was demonstrated with a mobile phone design case. Shieh et al. [22] advanced this methodology by incorporating a multi-objective evolutionary algorithm (MOEA) into FKES. Among the tested MOEAs, the non-dominated sorting genetic algorithm-II (NSGA-II) and the strength Pareto evolutionary algorithm-2 (SPEA2) excelled in convergence and diversity, respectively, as shown in a vase design case study. Wang [23] also adopted SVR within BKES, whereas he incorporated GST into FKES to accurately estimate the influence weighting of high-price machine tool shape parameters. In the high-speed train seat color study by Xie et al. [24], typical color schemes were generated via the practical color coordinate system. Within FKES, factor analysis and multidimensional scaling translated users’ emotional characteristics, while the analytic hierarchy process (AHP) and the independent weight coefficient method were employed for weighting. In BKES, the technique for order of preference by similarity to ideal solution (TOPSIS) optimized the color schemes, indicating that tones with medium brightness and saturation—light and soft—are preferable choices.

HKES features bi-directional translation, making it suitable for designing products that meet users’ emotional needs or preferences. In both FKES and BKES, the methodology applies consistent standards when selecting analytical tools and techniques. Specifically, the quantitative analysis of design factors in FKES should be based on users’ primary needs, and vice versa for the emotional or affective evaluation of design schemes in BKES [17,18,20]. This ensures that the selected designs closely match the users’ real-world contexts.

### Pleasure-arousal-dominance model

When people interact with product colors, visual and neural processing can subconsciously trigger emotional fluctuations and value judgments. These reactions occur through relevant emotional representations in the brain. At the same time, emotional signals that align with aesthetic preferences are generated. This reflects the brain’s integrated response to color perception [25,26]. Color functions both as a visual attribute and as an emotional medium. It is closely connected to environmental factors, cultural norms, and human behaviors. The way color is presented, and the emotional or affective responses it elicits, are strongly dependent on context [27,28].

Understanding how color’s emotional representation forms is essential for accurately assessing color experiences. Over time, many psychological measurement frameworks have been developed to analyze and categorize emotional responses in different contexts. These range from the James–Lange Theory of Emotion in the 1880s to the Constructed Emotion Theory in the 2000s [29, 30]. The PAD model, introduced by A. Mehrabian and J. Russell in the 1970s, scaled emotional states through three dimensions: pleasure-displeasure (P), arousal-nonarousal (A), and dominance-submissiveness (D) [30,31]. This model views human emotions as multidimensional psychological phenomena and has been widely used in emotional designs and related KE processes [32].

The PAD emotional scale is practical for evaluation and data processing. It is minimally influenced by external factors such as the environment and equipment. For this reason, it has been adopted in diverse fields including architecture, interior design, fashion, consumer goods, and electronics. Using the PAD scale and related theory, Tantanatewin et al. [33] and Divers [34] examined emotional responses to interior color in restaurant and therapeutic settings, respectively. Tantanatewin et al. found that pleasure was the strongest predictor of whether people would enter a restaurant, with bright and warm colors enhancing this feeling. Divers highlighted the nuanced effects of color value and chroma—beyond just hue—on emotions, advocating for an evidence-based approach to creating inviting environments. In rural accommodation lighting, Wei et al. [35] discovered that colored lighting combinations influenced arousal but had limited effects on pleasure and dominance. Warm white light combined with blue-yellow or green-yellow hues prominently boosted visual comfort and alleviated anxiety, while blue-red light increased attraction. Moreover, cool white light combinations could lessen the sensation of warmth.

Particularly, the Institute of Psychology at the Chinese Academy of Sciences developed a localized PAD scale to aid Chinese users. Using this scale, Gao et al. [36] and Zhao et al. [37] analyzed users’ emotional responses in Chinese Weibo texts and mobile library interfaces, respectively, confirming its applicability across various themes. Notably, Li et al. [31] reported that the Chinese version demonstrated reasonable reliability and validity in the P and D dimensions, but showed limitations in the A dimension.

### Fuzzy grey relation analysis

The targeted evaluation of design prototypes or schemes is essential in the user preference assessment phase. This step is also a key part of the BKES process. Current evaluation methods mainly rely on users’ emotion scales or custom Likert-scale questionnaires [38,39]. Users’ emotional responses to product features often involve randomness and uncertainty. Grey System Theory (GST), developed by Deng in the 1980s, was designed to analyze systems with uncertain or incomplete information. It is therefore a suitable mathematical approach for this context. In particular, Grey Relational Analysis—a widely used GST model—is commonly employed in emotional experiences and related KE processes [40,41]. It assesses the correlation between emotional responses and product features, refining designs by prioritizing features that evoke desired emotions.

Applying fuzzy weighting to grey relational degrees enhances the emotional preference assessment process. This combined method allows more nuanced decision-making in uncertain contexts. It also clarifies relationships between competing objectives. Researchers have extended this approach by combining GRA and fuzzy logic with other decision-making tools such as the Analytic Hierarchy Process (AHP), the Technique for Order Preference by Similarity to Ideal Solution (TOPSIS), and Quality Function Deployment (QFD).

Yadav et al. [42] introduced an integrative approach combining GRA, fuzzy AHP, and Taguchi methods to upgrade aesthetic quality in car profile design. By quantifying customer emotions and designer insights, they identified five key aesthetic attributes: originality, family-feeling, masculine, elegant, and modern. This approach effectively handled vague data, theoretically raising customer satisfaction and market competitiveness. Wang et al. successively conducted two studies using GRA and fuzzy logic to explore product evolution and user emotional experiences. Their GRA-fuzzy TOPSIS approach [43] captured consumer emotions and prioritized design alternatives, bridging cognitive gaps between consumers and designers, as demonstrated with smart capsule coffee machines. Additionally, their GRA-fuzzy QFD approach [44] mapped emotional needs to design parameters in wickerwork lamps, improving customer satisfaction and product distinction in competitive markets. Via combining fuzzy GRA and fuzzy AHP, Olabanji et al. [45] amended the conceptual design of pipe bending machines. This method evaluated feature weights and sub-features, comparing designs to an ideal benchmark, successfully identifying optimal solutions. Xue et al. [46] (Xue et al. 2019) and Zhou et al. [47] used a Kansei Engineering framework that incorporated fuzzy GRA to match the design elements of train seats and electric recliner chairs with user perceptions. Through 3D modeling and real-time evaluation based on relevant quantified design element matrix, both studies effectively predicted visualized product prototypes to match consumer imagery.

In summary, subjective evaluations in emotional design often contain ambiguity. Combining GRA with fuzzy sets helps to transform multi-objective evaluation metrics into manageable single-objective problems [45,47]. Specifically, GRA quantifies the similarity between referenced and evaluated emotional states, identifying emotional preferences and corresponding design features. Fuzzy sets offer a nuanced representation of users’ emotional preferences by effectively handling the vagueness inherent in their emotional responses. This synergy fosters more flexible and accurate evaluations, leading to more rational decision-making and raising the reliability of outcomes in complex environments.

### Application of eye-tracking technology in color analysis

Eye-tracking experiments capture a series of eye movement behaviors, such as fixations and saccades, which are elicited by visual perceptual stimuli and reflect changes in observers’ perceptual and cognitive demands. Eye-tracking technology records these ocular movements and provides quantitative physiological data that can be used to infer underlying cognitive processes and predict psychomotor performance. Owing to its ability to objectively reveal users’ perceptual and attentional patterns, eye-tracking has been widely applied in research on product appearance design [48], interface design [49], and landscape design [50].

Color variation is a critical visual stimulus that significantly influences eye movement behavior and visual attention allocation. Consequently, eye-tracking methods have been increasingly adopted in color-related design studies. For example, Lee et al. [51] employed eye-tracking technology to investigate the effects of different graphic designs on participants’ cognition, emotion, and perception, with particular emphasis on the role of color in emotional responses and coffee flavor perception. Pradhan et al. [52] explored the complex relationship between color psychology and consumer preferences in coffee packaging design. Yu et al. [53] combined eye-tracking data with subjective evaluations to examine preferences for rosewood and wenge wood under different hue and lightness conditions. Hu et al. [54] integrated eye-tracking techniques with questionnaire surveys to analyze the relationships between color and visual perception in historical museum exhibition environments, focusing on hue, saturation, and brightness.

Overall, these studies demonstrate that eye-tracking technology provides a powerful and objective means for analyzing color perception and visual attention. In this study, eye-tracking is therefore adopted as an independent validation approach to support the analysis of color emotional evaluation results.

## Methods and framework

Based on the guideline of HKES, we integrated the PAD model and fuzzy GRA in the approach for our product color emotional design. The proposed approach comprises four stages, with Stage 1 and Stage 2 serving as the primary stages:

1) Color Emotional Design: Developing color design schemes using the PAD emotional scaling.
2) User Preference Assessment: Evaluating and optimizing design schemes through fuzzy GRA.

**Fig 1:**
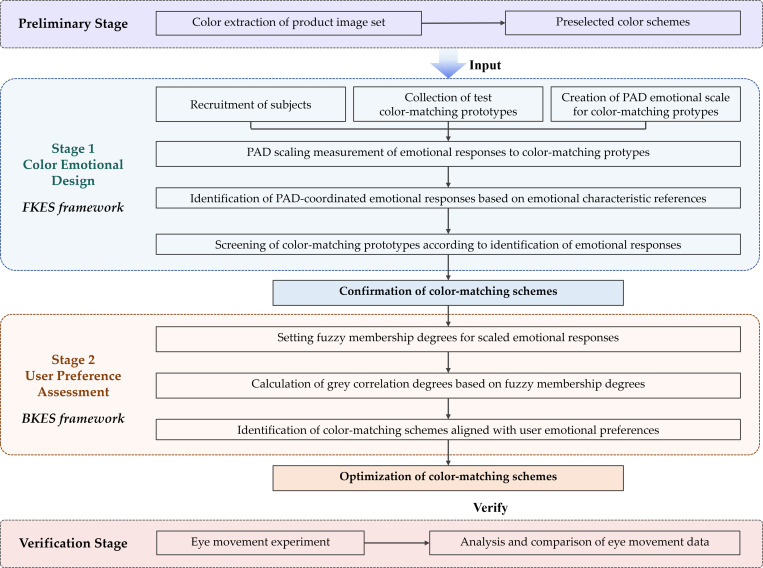
Detailed flow of the research approach.

Stage 1 follows the FKES framework, while Stage 2 operates within the BKES framework. The detailed approach is illustrated in Fig 1.

### Preliminary stage: extraction of product color schemes

**Fig 2:**
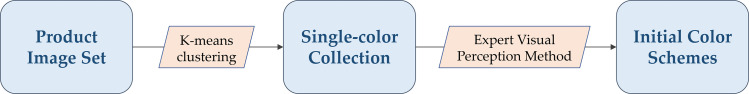
Process and methods for extracting initial product color schemes.

Before conducting the formal Kansei Engineering analysis, it is necessary to first extract product colors and construct representative color schemes, which serve as the basis for subsequent evaluation and measurement. In this study, an initial screening of product color schemes was performed by combining k-means clustering with expert visual assessment. The overall procedure is illustrated in Fig 2.

The process begins with the extraction of representative colors from existing product images. After removing background elements and mitigating illumination interference, the k-means clustering algorithm is independently applied to each product image to identify visually dominant color components. To ensure mathematical rigor in the color extraction process, the k-means clustering method adopted in this study aims to minimize the within-cluster sum of squared errors, which can be expressed as follows [55]:


$$\underset{\left\{\mu_{\text{1}},\mu_{\text{2}},\ldots ,\mu_{k}\right\}}{\text{min}}\sum_{i=\text{1}}^{k}\sum_{x\in C_{i}}{\left\|x-\mu_{i}\right\|}^{\text{2}}$$ (1)

Here, *x* denotes a pixel in the product image represented as a vector in the color space; *C*_*i*_ represents the *i*-th cluster; *μ*_*i*_ is the centroid color of cluster *C*_*i*_; and *k* is the number of clusters. The k-means algorithm partitions image pixels into multiple regions by iteratively minimizing intra-cluster variance, grouping pixels with similar color attributes together. Each cluster is represented by a centroid, which can be regarded as a representative single color reflecting a major component of the product’s appearance.

Considering the characteristics of product color composition and to reduce the influence of highlights and shadows, the number of clusters was set to 5 during the color extraction stage. In practice, the k-means clustering was implemented in a Python environment using standard clustering functions to process pixel-level image data. Upon convergence, the algorithm outputs the centroid colors of each cluster along with their corresponding pixel proportions. These centroid colors are treated as representative single colors for subsequent screening and color scheme construction.

For each product, the extracted colors are ranked according to their pixel proportions, and the top-ranked colors are retained as primary single-color candidates. Subsequently, all extracted single colors from different products are matched to the closest Pantone color references to form a unified single-color library. Based on this color library, a group of experts with backgrounds in product design and color design were invited to combine the extracted single colors into feasible two-color and three-color schemes, following criteria of color harmony and suitability for the product. Through expert discussion and evaluation, a set of representative color schemes was finally selected and used as the base schemes for subsequent PAD-based evaluation and analysis.

### Stage 1: color emotional design

This stage primarily involved creating the PAD emotional scale and measuring, analyzing, and screening color-matching prototypes based on the scale. Each dimension—P, A, D—contained four pairs of opposite adjectives, listing from V_1_ to V_12_, as displayed in Table 1. These pairs applied a nine-point scale from −4 to +4 to describe users’ emotional responses [56]. Through scaling these responses along the adjective axes, each emotion is mapped to a specific PAD coordinate. For color design, this three-dimensional description visualizes how different colors evoke emotions, helping identify those that elicit positive responses [30]. By inserting measurement results into Equation system (2), the actual PAD coordinate (*P*, *A*, *D*) for a test prototype’s emotional response is obtained.

**Table 1:** PAD emotion scale with 12 pairs of opposite adjectives.

	P		A		D
**V**_**1**_	Angry-Interested	**V**_**2**_	Awake-Sleepy	**V**_**3**_	Controlled-Master
	○-4○-3○-2○-1○0○1○2○3○4		○-4○-3○-2○-1○0○1○2○3○4		○-4○-3○-2○-1○0○1○2○3○4
**V**_**4**_	Friendly-Dismissive	**V**_**5**_	Calm-Excited	**V**_**4**_	Dominant-Submissive
	○-4○-3○-2○-1○0○1○2○3○4		○-4○-3○-2○-1○0○1○2○3○4		○-4○-3○-2○-1○0○1○2○3○4
**V**_**7**_	Miserable-Happy	**V**_**8**_	Interested-Relaxed	**V**_**7**_	Humble-Arrogant
	○-4○-3○-2○-1○0○1○2○3○4		○-4○-3○-2○-1○0○1○2○3○4		○-4○-3○-2○-1○0○1○2○3○4
**V**_**10**_	Excited-Irritated	**V**_**11**_	Stiff-Surprised	**V**_**12**_	Influential-Affected
	○-4○-3○-2○-1○0○1○2○3○4		○-4○-3○-2○-1○0○1○2○3○4		○-4○-3○-2○-1○0○1○2○3○4


$$\left\{ \begin{array}{c} P=\frac{V_{\text{1}}-V_{\text{4}}+V_{\text{7}}-V_{\text{1}\text{0}}}{\text{4}}\\ A=\frac{-V_{\text{2}}{+V_{\text{5}}-V_{\text{8}}+V}_{\text{1}\text{1}}}{\text{4}}\\ D=\frac{V_{\text{3}}-V_{\text{6}}+V_{\text{9}}-V_{\text{1}\text{2}}}{\text{4}} \end{array}\right.\;$$ (2)

Furthermore, ten distinct color-perception-linked emotional characteristics—five positives and five negatives—were selected from previous studies [20,34,56,57]. As exhibited in Table 2, these emotional states and their PAD coordinates served as reference points for assessing PAD-coordinated emotional responses and corresponding color schemes. As is known, emotional discrepancy refers to the absolute distance between the participates’ actual emotional response and the reference emotion in the PAD dimensions. The minimal distance, calculated using Equation (3), indicates how closely the participates’ emotional state aligns with the reference, revealing the specific emotional characteristic evoked by a color-matching prototype [20,56,57].

**Table 2:** 10 adopted emotion characteristic references and their PAD coordinates.

No.	Emotion	Description	P-value (p_n_)	A-value (a_n_)	D-value (d_n_)
**01**	**Positive**	Joyful	2.77	1.21	1.42
**02**		Relaxed	2.19	−0.66	1.05
**03**		Surprised	1.72	1.71	0.22
**04**		Dependent	0.39	−0.81	−1.48
**05**		Mild	1.57	−0.79	0.38
**06**	**Negative**	Bored	−0.53	−1.25	−0.84
**07**		Sad	−0.89	0.17	−0.70
**08**		Anxious	−0.95	0.32	−0.63
**09**		Contemptuous	−1.58	0.32	1.02
**10**		Disgusted	−1.80	0.40	0.67


$$L_{mn}=\sqrt{{\left(P_{m}-p_{n}\right)}^{\text{2}}+{\left(A_{m}-a_{n}\right)}^{\text{2}}+{\left(D_{m}-d_{n}\right)}^{\text{2}}}\;\;(\text{m}\in \{\text{1},\;\text{2},\;\ldots ,\;\text{2}\text{0}\},\;\;\text{n}\in \{\text{1},\;\text{2},\;\ldots ,\;\text{1}\text{0}\})$$ (3)

Here, *L*_*mn*_ represents the emotional discrepancy between an actual emotional response and each emotional characteristic (No. *n*) for each color-matching prototype (No. *m*). The variables *m* and *n* refer to the specific color-matching prototype and emotion characteristic reference, respectively. Meanwhile, (*P*_*m*_, *A*_*m*_, *D*_*m*_) and (*p*_*n*_, *a*_*n*_, *d*_*n*_) denote the PAD coordinates for the corresponding prototype’s emotional response and the specific reference, respectively.

The minimum value, *L*_*mn*-min_, within the result set [*L*_*mn*_] reveals the emotional characteristic of the prototype. After identifying and eliminating prototypes likely to evoke negative emotions, the remaining color-matching schemes were prepared for further optimization. To empirically validate this stage, participants were recruited to evaluate the color-matching prototypes. Measurements were collected from 216 participants during the recruitment period from 10/10/2024–25/11/2024.

### Stage 2: user preference assessment

Stage 2 primarily focused on the targeted assessment and optimization of the emotional preferences associated with the remaining color-matching schemes. Additionally, to upgrade qualitative analysis, an extra dimension—user satisfaction (S)—was introduced into the PAD coordinate system. For four-dimensional PADS emotional responses, the fuzzy GRA calculation and assessment process can be divided into the following steps [45,56,58]:

1) Establishing comparison “emotion” matrix and reference sequence, normalizing original “emotion” data.
2) Computing fuzzy membership degrees, determining absolute differences between normalized “emotion” values and grey relational coefficients.
3) Calculating fuzzy grey relational degrees.

In the first step, all remaining schemes’ PADS-coordinated emotional responses were integrated into a comparison matrix *X*, as displayed below:


$$X=[\begin{array}{c} \begin{array}{c} x_{P}(i)\\ x_{A}(i)\\ x_{D}(i) \end{array}\\ x_{S}(i) \end{array}]=[\begin{array}{ccc} x_{P}(\text{1}) & x_{P}(\text{2}) & \begin{array}{cc} \cdots & x_{P}(\text{1}\text{0}) \end{array}\\ x_{A}(\text{1}) & x_{A}(\text{2}) & \begin{array}{cc} \cdots & x_{A}(\text{1}\text{0}) \end{array}\\ \begin{array}{c} x_{D}(\text{1})\\ x_{S}(\text{1}) \end{array} & \begin{array}{c} x_{D}(\text{2})\\ x_{S}(\text{2}) \end{array} & \begin{array}{c} {\cdots \;\;\;\;x}_{D}(\text{1}\text{0})\\ \begin{array}{cc} \cdots & x_{S}(\text{1}\text{0}) \end{array} \end{array} \end{array}](xu’(t)\in X,\;\text{u}\in \{\text{P},\text{A},\text{D},\text{S}\},\;\text{i}\in \{\text{1},\;\text{2},\ldots ,\;\text{10}\})$$ (4)

Due to the vagueness in emotions and their evaluations, min-max normalization ensured all evaluation data fell within the [0,1] range, enhancing computability. As shown in Equation (5) and (6), *x*_*u*_*’(i)*, *X* were normalized from *x*_*u*_*(i)*, X, respectively.


$$x_{u}^{\prime }(i)=\frac{x_{u}(i)-{x_{u}}_{min}}{{x_{u}}_{max}-{x_{u}}_{min}}$$ (5)

$$X^{\prime }=\left[x_{u}^{\prime }(i)\right]=[\begin{array}{c} \begin{array}{c} x_{P}^{\prime }(i)\\ x_{A}^{\prime }(i)\\ x_{D}^{\prime }(i) \end{array}\\ x_{S}^{\prime }(i) \end{array}]$$ (6)

In the second step, a fuzzy similarity matrix was constructed based on X’ using the cosine angle method, followed by the calculation of fuzzy membership degrees [45,58]:


$$r_{u}=\frac{\sum_{i=\text{1}}^{\text{1}\text{0}}x_{u}^{\prime }(i){x_{u}^{\prime }}_{max}}{\sqrt{\sum_{i=\text{1}}^{\text{1}\text{0}}x_{u}^{{}^{\prime }\text{2}}(i)}\sqrt{\sum_{i=\text{1}}^{\text{1}\text{0}}{x_{u}^{{}^{\prime }\text{2}}}_{max}}}$$ (7)

Here, *r*_*u*_ represented the fuzzy membership degree for each remaining scheme, *x’_umax_* referred to the optimal emotional response within *x*_*u*_*(i)* in each emotional dimension.

Next, the absolute differences of *x*_*u*_*’(i)* were calculated as follows:


$$\Delta x_{u}^{\prime }(i)=\left|x_{u}^{\prime }(i)-{x_{u}^{\prime }}_{max}\right|$$ (8)

$${\Delta X}^{\prime }=\left[{\Delta x}_{u}^{\prime }(i)\right]==[\begin{array}{c} \begin{array}{c} {\Delta x}_{P}^{\prime }(i)\\ {\Delta x}_{A}^{\prime }(i)\\ \Delta x_{D}^{\prime }(i) \end{array}\\ \Delta x_{S}^{\prime }(i) \end{array}]$$ (9)

The maximum absolute difference, *Δx’_max_*, and the minimum, *Δx’_*min*_*, among *ΔX’* were used in Equation (10) to work the grey relational coefficient out. To strengthen the distinction between obtained coefficients, the resolution coefficient, *ρ*, was typically set to 0.5 [58,59].


$$\delta_{u}(i)=\frac{{\Delta x}_{min}^{\prime }+\rho \Delta x_{max}^{\prime }}{\Delta X_{u}(i)+\rho \Delta x_{max}^{\prime }}$$ (10)

Since grey relational coefficients were based on absolute differences between comparison and reference sequences, they were fairly dispersed, complicating overall comparison. Therefore, when calculating the relational degree, these coefficients were averaged into a single value.


$$R_{u}=\frac{\text{1}}{\text{2}}\left[\frac{\sum_{i=\text{1}}^{\text{1}\text{0}}x_{u}^{\prime }(i){x_{u}^{\prime }}_{max}}{\sqrt{\sum_{i=\text{1}}^{\text{1}\text{0}}x_{u}^{{}^{\prime }\text{2}}(i)}\sqrt{\sum_{i=\text{1}}^{\text{1}\text{0}}{x_{u}^{{}^{\prime }\text{2}}}_{max}}}+\frac{\text{1}}{\text{1}\text{0}}\sum_{i=\text{1}}^{\text{1}\text{0}}\delta_{u}(i)\right]$$ (11)

With the fuzzy grey relational degree, *Ru*, obtained using Equation (11), color-matching schemes could be ranked and screened according to their fuzzy grey relational degrees.

### Verification stage: eye-tracking experiment

To validate the rationality and accuracy of the proposed HKES approach in color scheme evaluation, an eye-tracking experiment was conducted to objectively examine the consistency between the model-based ranking results and users’ actual visual behavior. The verification procedure included experimental design, eye-tracker calibration, and eye-tracking data collection and analysis.

Attention heatmaps were used to visualize the spatial distribution of visual attention across different color schemes, providing an intuitive indication of their relative visual attractiveness. [60] However, as heatmaps primarily reflect overall attention patterns and are limited in quantitative comparison, total fixation duration was further adopted as a key eye-tracking metric. This indicator represents the cumulative time participants fixated on a given stimulus and is widely associated with visual interest and preference [61].

By combining attention heatmaps and total fixation duration, the visual attention characteristics of different color schemes were analyzed and compared with the rankings obtained from the HKES model. The consistency between eye-tracking results and model-based evaluations provides additional evidence supporting the effectiveness and cognitive plausibility of the proposed HKES approach.

The experiment was conducted according to the guidelines of the Declaration of Helsinki and approved by the Institutional Ethics Committee of Anhui University (protocol code: BECAHU-2024–014).

## Case research and results

### Color scheme extraction and participants recruitment

In this case study, a typical household hair dryer with diverse color designs was selected as the research object. Product images were collected from online retail platforms, brand official websites, and live-streaming channels. A total of 36 representative product images were selected as samples for color extraction. Following the color extraction procedure established in Section 3.1, k-means clustering was applied to process the color information of the product images. Each product image was analyzed independently to extract its primary visual color components, and the extracted results were subsequently aggregated and screened. As a result, an initial set of 36 × Top 2 = 72 single-color candidates was obtained.

Subsequently, these candidate colors were standardized and matched to Pantone color references. Single colors with highly similar color attributes were merged, resulting in a unified single-color library consisting of 25 representative colors. This color library effectively reflects the mainstream color characteristics commonly observed in household hair dryer products.

Based on the constructed single-color library, six experts with backgrounds in product design and color design were invited to participate in the color scheme construction through offline group discussions. Guided by principles of color harmony and the practical application characteristics of household hair dryers, the experts combined the single colors to generate initial two-color and three-color schemes. During the scheme construction process, minor adjustments to individual colors were permitted based on conventional color-matching guidelines, such as warm-cool color balance and the coordination of lightness and saturation, in order to enhance overall visual harmony and product suitability. Through multiple rounds of visual evaluation and group discussion, schemes that did not align with the product’s aesthetic characteristics or practical applicability were gradually eliminated. Ultimately, 20 representative product color schemes were selected as the input for subsequent PAD-based emotional dimension evaluation, enabling analysis of the effects of different color combinations on users’ emotional experiences.

**Fig 3:**
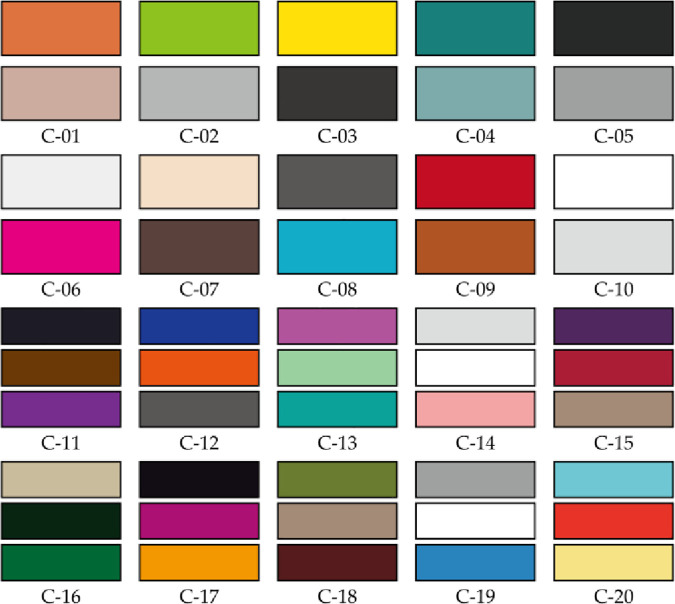
10 dual- and 10 tri-color test cases applied to color-matching prototypes during Stage 1.

The 20 suitable color schemes were categorized into dual-color and tri-color combinations. In the dual-color schemes, each design consisted of a primary and a secondary color. The tri-color schemes included a primary, secondary, and accent color. As depicted in Fig 3, cases C-01 to C-10 are dual-color, and C-11 to C-20 are tri-color. Each category contained 10 samples covering cool, warm, and neutral tones, presented on visual test boards for subjective evaluation.

To ensure the validity of the color PAD emotional experience test, participants needed familiarity with hair dryer selection and usage, as well as color perception and cognition. Participants were selected based on three criteria: 50% male and female distribution, ages 21–50 and generally healthy with normal corrected vision, free from color blindness or other ocular conditions. Ultimately, 216 participants were chosen, equally split by gender.

### Emotional experience of color-matching case

Based on the case sample set and the Chinese version of the PAD emotional experience scale, we developed a Likert scale questionnaire to assess participants’ emotional responses to the hair dryer color-matching samples. The complete questionnaire is provided in S1 File. The questionnaire was conducted via an online survey. Participants remotely rated standardized color-matching stimuli (dual-color and tri-color schemes) using Likert scales displayed on the questionnaire interface. Participants were recruited during the period from 10/10/2024–25/11/2024. A total of 216 individuals completed the survey. After removing 8 incomplete questionnaires with missing or incorrect selections, the effective response rate was 96.296%, resulting in valid measurement data of 208 × 20 × 12, totaling 49,920 data points.

After importing the data into SPSS 23.0 for reliability testing, a Cronbach’s alpha of 0.796 was revealed, indicating satisfactory reliability for further analysis. We then calculated the PAD mean values for each of the 20 cases (as shown in Table 3). These mean values were substituted into Equation (2) to obtain the actual PAD measurements for each case. One-way ANOVA was conducted on the PAD emotional ratings obtained from the survey to examine whether different color schemes elicited statistically significant differences in emotional responses. The results showed significant main effects of color scheme on pleasure (P: p = 0.017 < 0.05), arousal (A: p = 0.048 < 0.05), and dominance (D: p = 0.031 < 0.05), indicating that the proposed color combinations produced distinguishable emotional experiences across these three dimensions. By comparing these measurements with the PAD benchmark coordinates in Table 2 using Equation (3), we determined the proximity of each sample’s emotional response to the reference emotional characteristics, as presented in Table 4. The data marked with an asterisk in Table 4 indicate the PAD emotional characteristics of the 20 color schemes.

**Table 3:** Mean statistics from PAD scaling measurements of color-matching case samples during Stage 1.

Case No.	P				A				D			
	V_1_	V_4_	V_7_	V_10_	V_2_	V_5_	V_8_	V_11_	V_3_	V_6_	V_9_	V_12_
C-01	−1.212	0.755	0.553	1.163	0.904	−1.601	1.245	−1.716	−0.596	1.303	−0.861	0.207
C-02	2.072	−1.346	2.197	−2.279	0.144	−1.197	0.404	−0.803	1.558	−0.058	0.798	−2.125
C-03	1.851	−1.514	1.899	−1.688	−2.048	1.351	−1.654	1.798	1.149	−0.048	0.255	−0.101
C-04	0.149	0.755	−1.048	1.596	−0.096	0.058	−1.255	−0.649	0.601	0.351	−0.942	1.558
C-05	0.852	−0.188	0.505	0.399	1.899	−1.851	−0.702	0.197	−1.197	0.649	−1.702	1.303
C-06	1.534	−1.764	2.413	−2.442	−0.947	2.351	−0.904	0.548	1.731	−1.226	1.308	−1.385
C-07	−1.505	1.899	−0.649	−1.702	1.048	−2.048	1.851	0.495	−0.947	0.298	−2.101	0.755
C-08	0.601	0.399	2.149	0.452	0.962	−1.346	1.899	0.755	−1.202	0.202	−2.351	1.798
C-09	−1.159	1.106	−0.837	0.548	−1.452	−0.798	−0.553	−0.154	−0.409	0.452	−0.082	0.755
C-10	2.255	−1.135	0.952	−2.308	1.899	−1.303	0.048	−0.202	0.649	−1.755	−0.048	0.404
C-11	−0.505	0.096	−2.303	−0.452	0.702	−1.755	1.144	−0.952	−0.149	1.601	−1.351	0.851
C-12	0.303	−2.048	1.798	−2.351	−1.601	2.048	−0.856	2.245	−0.538	1.163	0.442	−1.909
C-13	−1.803	0.452	−1.399	2.096	−0.087	1.221	−0.688	−0.346	1.034	−0.587	0.856	−1.173
C-14	2.447	−1.755	1.846	−2.303	−1.442	0.601	−1.202	1.505	−0.087	−2.245	1.543	−1.952
C-15	−1.207	1.361	−2.255	1.875	−0.913	0.192	−0.452	0.144	1.601	−1.149	1.255	1.452
C-16	−1.053	0.048	−0.899	0.101	0.197	−1.798	1.298	−1.558	−0.101	1.659	−1.495	0.798
C-17	1.245	−1.851	1.899	−1.755	−2.082	1.755	−1.351	1.962	−0.308	−1.149	0.048	−0.264
C-18	−2.255	1.106	−1.865	1.875	0.303	0.851	−0.755	0.548	0.697	−0.952	0.351	−1.053
C-19	1.495	−1.048	1.606	−1.702	1.255	−0.548	1.144	−0.505	0.202	1.101	−0.154	−1.952
C-20	−0.606	1.255	−1.899	1.793	−0.505	0.635	−0.351	0.159	1.452	−1.505	1.745	1.149

**Table 4:** PAD-coordinated values and emotional characteristics closeness of color-matching prototypes.

Case No.	Actual PAD coordinates			User’s emotional character tendency of PAD Model									
				Positive emotions					Negative emotions				
	P_m_	A_m_	D_m_	Joyful	Relaxed	Surprised	Dependent	Mild	Boring	Sadness	Anxiety	Contempt	Disgust
C-01	−0.643	−1.367	−0.742	4.791	3.424	3.996	1.385	2.546	0.189*	1.562	1.719	2.614	2.542
C-02	1.974	−0.637	1.135	2.031	0.233*	2.532	3.062	0.870	3.248	3.496	3.547	3.682	3.941
C-03	1.738	1.713	0.388	1.544	2.505	0.169*	3.416	2.509	3.928	3.236	3.194	3.654	3.784
C-04	−0.813	0.188	−0.563	4.221	3.513	3.057	1.812	2.743	1.492	0.158*	0.202	1.764	1.594
C-05	0.288	−0.713	−1.213	4.098	2.957	3.158	0.302*	2.046	1.047	1.559	1.715	3.089	3.024
C-06	2.038	1.188	1.413	0.732*	1.889	1.340	3.883	2.280	4.197	3.752	3.722	3.741	3.988
C-07	−0.588	−1.113	−1.025	4.759	3.497	3.853	1.120	2.595	0.237*	1.358	1.530	2.687	2.575
C-08	0.475	−0.863	−1.388	4.177	2.988	3.280	0.136*	2.081	1.208	1.845	2.001	3.379	3.318
C-09	−0.913	0.263	−0.425	4.227	3.558	3.073	1.991	2.815	1.615	0.291	0.216*	1.593	1.416
C-10	1.663	−0.863	0.488	2.528	0.797	2.588	2.344	0.160*	2.593	2.999	3.079	3.493	3.691
C-11	−0.613	−1.138	−0.988	4.770	3.498	3.875	1.164	2.600	0.203*	1.368	1.539	2.663	2.554
C-12	1.625	1.688	0.163	1.766	2.573	0.113*	3.235	2.488	3.779	3.062	3.022	3.589	3.694
C-13	−1.438	0.413	0.913	4.313	3.786	3.484	3.250	3.283	2.581	1.721	1.621	0.201*	0.436
C-14	2.088	1.188	1.413	0.682*	1.886	1.353	3.904	2.291	4.228	3.791	3.763	3.790	4.036
C-15	−1.675	0.425	0.638	4.581	4.035	3.654	3.206	3.475	2.510	1.572	1.464	0.407	0.131*
C-16	−0.525	−1.213	−1.013	4.759	3.454	3.886	1.104	2.551	0.177*	1.464	1.636	2.756	2.657
C-17	1.688	1.788	0.288	1.669	2.613	0.108*	3.400	2.582	3.927	3.200	3.155	3.657	3.773
C-18	−1.775	0.463	0.763	4.653	4.131	3.750	3.367	3.592	2.656	1.735	1.625	0.353	0.115*
C-19	1.463	−0.863	0.225	2.726	1.118	2.586	2.015	0.202*	2.293	2.731	2.820	3.360	3.527
C-20	−1.388	0.413	0.888	4.267	3.739	3.433	3.204	3.233	2.547	1.682	1.583	0.251*	0.466

The results indicated that both dual-color and tri-color schemes could evoke positive and negative emotions, suggesting no conspicuous difference in emotional preference based on the number of colors. However, dual-color schemes were more likely to evoke positive emotions in 6 cases, compared to 4 for tri-color schemes, indicating a user preference for dual-color designs. Overall, 50% of the color schemes elicited positive emotions, demonstrating that current market designs align with user preferences but have room for improvement. Specific dual-color cases (C-01, C-04, C-07, C-09) and tri-color cases (C-11, C-13, C-15, C-16, C-18, C-20) were more likely to evoke negative emotions such as boredom, sadness, anxiety, contempt, and disgust. Issues included the use of similar adjacent colors, overly bright or dark tones, and mismatched styles with the product’s function. Adjustments in color choices, particularly avoiding overly monotonous or contrasting schemes, could enhance user satisfaction.

To optimize the emotional design of hair dryer colors, we excluded 10 color schemes that tended to evoke negative emotions. We then conducted a PAD emotional analysis on color schemes that elicited positive emotions. This analysis, informed by design principles and participant feedback, provides insights for designing emotionally appealing products.

For the dual-color schemes, six cases (C-02, C-03, C-05, C-06, C-08, and C-10) were identified as positive designs. Among them, C-02 and C-03 adopted bright tones such as green and yellow, which effectively enhanced user pleasure and attention, though slight reductions in saturation could further balance excitement levels. In contrast, C-05 and C-10 relied on neutral combinations of gray and white, generating feelings of calmness and comfort, with increased brightness expected to improve user interest. Meanwhile, C-06 and C-08 combined magenta with blue to create a more harmonious and balanced appearance, where adjustments in brightness could contribute to a more uplifting overall mood.

For the tri-color schemes, four cases (C-12, C-14, C-17, and C-19) were identified as positive designs. Specifically, C-12 and C-17 employed contrasting colors such as blue and orange, which successfully captured attention and evoked a sense of surprise, though further adjustments in brightness and saturation could help achieve a more balanced emotional response. C-14 combined gray, white, and pink to produce a joyful effect, with an increase in the brightness of gray recommended to avoid visual monotony. In contrast, C-19 applied neutral tones accented with cool colors, generating a soothing and comfortable impression, and enhancing the accent color would likely strengthen its emotional impact. This analysis shows that both dual-color and tri-color schemes can effectively cater to diverse emotional preferences, offering guidance for future color design strategies.

### User preference assessment of color-matching schemes

**Fig 4:**
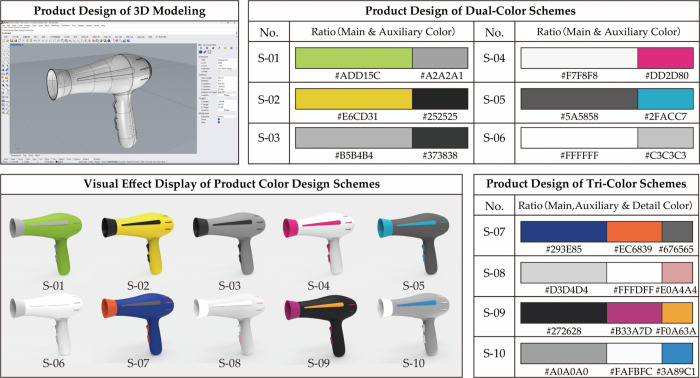
Confirmed color-matching schemes for Stage 2.

During this stage, we used previous positive emotional color schemes as references, focusing on dual-color and tri-color combinations. Dual-Color Schemes included 6 designs (S-01 to S-06) featuring main colors like yellow and green, with magenta and blue as accents. Each design incorporates varying proportions of neutral tones. Tri-Color Schemes included 4 designs (S-07 to S-10) inspired by “Pop Art” and “Minimalism.” These use ultramarine and black as main colors, with orange-red and purple as accents, and gray and yellow as highlights. Minimalist designs use black, white, and gray with pink and cobalt blue as accents. The color renderings were created using KeyShot software, resulting in 10 color-matching schemes for hair dryers (refer to Fig 4, with all colors using the HSV model [62]). This completes Stage 1 based on the FKES framework.

The BKES framework was utilized to assess the PAD emotional responses associated with ten hair dryer color design schemes. The evaluation questionnaire encompassed four dimensions: Pleasure (P), Arousal (A), Dominance (D), and Satisfaction (S), each rated on a scale from −4 (“strongly disagree”) to 4 (“strongly agree”). Concurrently, following the PAD emotional experience assessment, participants were asked to provide subjective evaluations of their visual preferences concerning the product’s color design. This evaluation metric, termed Subjective Visual Preferences (V), was consistent with the dimensions employed in the PAD assessment scale. The complete questionnaire is provided in S2 File.

Considering the requirement for collecting a relatively large number of questionnaire responses, the emotional experience evaluation was conducted via an online questionnaire survey. A total of 216 questionnaires were distributed, yielding 212 valid responses and resulting in an effective response rate of 98.148%. This provided a valid dataset comprising 212 × 10 × 4 and 212 × 10, totaling 10,600 data points. The data was imported into SPSS 23.0 for reliability testing, which revealed a Cronbach’s alpha of 0.817, indicating good structural reliability for further analysis. Subsequently, the average scores for each design scheme were calculated (Table 5), and one-way ANOVA was conducted on the PAD emotional ratings obtained from the second questionnaire survey to further examine whether the ten color schemes elicited statistically significant differences in emotional responses. The results revealed significant main effects of color scheme on pleasure (P: p = 0.008 < 0.05), arousal (A: p = 0.017 < 0.05), dominance (D: p = 0.035 < 0.05), and satisfaction (S: p = 0.026 < 0.05). Fuzzy Grey Relational Analysis was conducted using formulas (4) to (10). This analysis determined the fuzzy membership and grey relational degrees for each scheme. These results were applied to formula (11) to compute the fuzzy grey relational degree, facilitating the prioritization of the color design schemes. Additionally, after the fuzzy grey relational analysis, the mean values of subjective visual preference evaluations were normalized to further prioritize the color design schemes based on subjective assessments, as shown in Table 7. The resulting order of preference was S-06, S-05, S-04, S-07, S-01, S-03, S-08, S-10, S-02, S-09.The emotional experience evaluation of hair dryer color design schemes, based on fuzzy grey relational analysis, resulted in the following priority order (Table 7 and Fig 4): S-06, S-05, S-04, S-01, S-07, S-08, S-03, S-10, S-02, S-09.

**Table 5:** Mean statistics of PAD-coordinated emotional responses in Stage 2.

Scheme No.	User emotional experience of comprehensive evaluation index			
	P	A	D	S
S-01	1.014	0.736	1.566	1.575
S-02	−0.392	−0.302	0.146	0.198
S-03	0.297	−0.052	0.354	0.651
S-04	1.505	1.745	1.882	2.104
S-05	1.807	1.245	2.052	2.396
S-06	2.495	1.349	1.948	2.849
S-07	0.703	0.665	1.349	1.547
S-08	0.552	0.929	1.099	0.981
S-09	−0.203	0.165	−0.297	−0.604
S-10	0.057	−0.443	0.245	0.495

**Table 7:** Subjective visual preferences of Mean statistics & Normalized results in Stage 2.

Subjective Visual Preferences	Scheme No.									
	S-01	S-02.	S-03.	S-04.	S-05.	S-06.	S-07.	S-08.	S-09.	S-10
Mean Statistics	1.594	0.358	1.118	2.146	2.462	2.825	1.703	0.929	0.146	0.519
Normalized Results	0.541	0.079	0.363	0.747	0.865	1.000	0.581	0.292	0.000	0.139
Prioritization	5	9	6	3	2	1	4	7	10	8

The top-ranked schemes, S-06, S-05, S-04, S-01, and S-07, emerged as the preferred options from this evaluation. Additionally, the root mean square error (RMSE) was computed between the fuzzy grey relational degrees and the normalized subjective visual preferences for the ten color design schemes in Table 6 and Table 7. An RMSE value of 0.111, which is close to 0, indicates the reliability of both subjective and objective assessment results, affirming the credibility of the scheme prioritization. This concludes Stage 2 of the BKES framework, establishing a research framework for product color emotional design that integrates mixed emotional engineering with PAD emotional measurement and fuzzy grey relational analysis.

**Table 6:** Results of fuzzy grey correlation analysis of color-matching schemes in Stage 2.

Scheme No.	Fuzzy membership degree	Grey relational grade	Fuzzy grey relational grade	Prioritization
S-01	0.982	0.574	0.778	4
S-02	0.792	0.364	0.578	9
S-03	0.969	0.406	0.687	7
S-04	0.986	0.777	0.881	3
S-05	0.994	0.789	0.891	2
S-06	0.997	0.913	0.955	1
S-07	0.977	0.536	0.756	5
S-08	0.973	0.508	0.741	6
S-09	0.602	0.356	0.479	10
S-10	0.833	0.381	0.607	8

**Fig 5:**
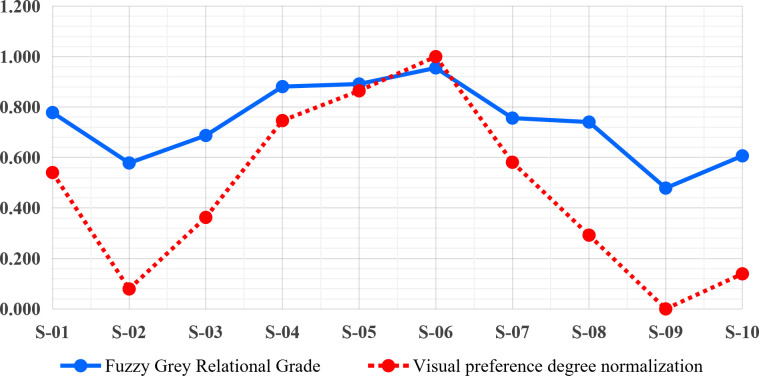
Subjective and objective prioritization of color-matching schemes in Stage 2.

Moreover, Fig 5 illustrates the comparison between subjective and objective prioritization results for the ten color-matching schemes in Stage 2. The horizontal axis represents the different schemes (S-01 to S-10), while the vertical axis shows their relative prioritization values. The dark bars correspond to the fuzzy grey relational degrees (objective evaluation), and the light bars represent the normalized results of subjective visual preference assessments. As shown in the figure, both evaluation perspectives reveal a consistent preference order, with schemes S-06, S-05, and S-04 ranking highest, followed by S-01 and S-07. The strong alignment between subjective and objective prioritization further validates the reliability of the proposed evaluation framework, confirming that the integrated PAD–fuzzy GRA approach can accurately capture users’ emotional preferences in color design.

### Eye-tracking experimental verification

To ensure the objectivity and feasibility of the eye-tracking experiment, a total of 24 participants were recruited for the color imagery cognition experiment, including 12 males and 12 females aged between 21 and 50 years. All participants were in good physical condition, had normal or corrected-to-normal vision, and reported no color blindness, color weakness, or other ocular diseases.

The eye-tracking experiment was conducted using a Tobii Pro X3-120 eye tracker in combination with a desktop computer. The device accurately records participants’ gaze positions and has a sampling rate of 120 Hz. The eye tracker measures 324 mm (12.7 inches) in length, weighs 118 g (4.2 oz.), and can be mounted directly onto computer monitors up to 25 inches using the provided magnetic mounting bracket. The experimental computer was equipped with a Windows 10 (64-bit) operating system, an Intel Core™ i7-14700 CPU, and 32 GB of RAM. The display resolution was set to 1920 × 1080 in full-screen mode.

**Fig 6:**
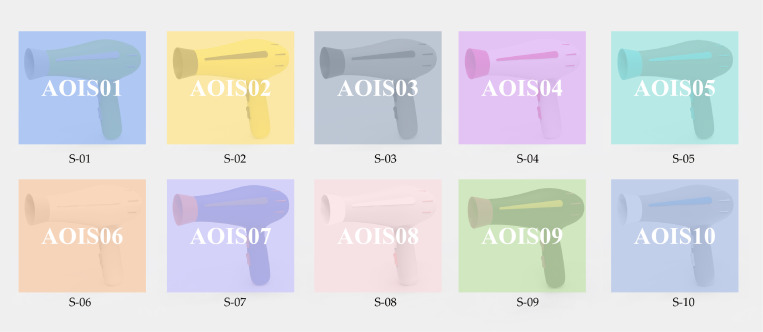
Areas of interest (AOIs) for experimental samples.

Prior to stimulus presentation, areas of interest (AOIs) were defined to ensure the validity of the collected eye-tracking data. In the visual test panel, a total of ten AOIs were delineated, each corresponding to one color scheme sample, as shown in Fig 6.

**Fig 7:**
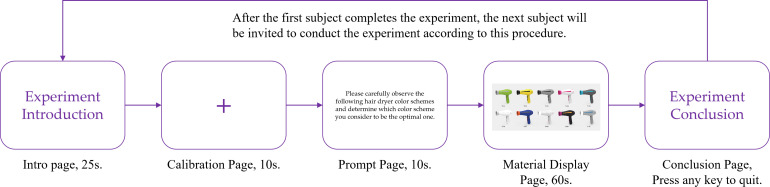
Eye-tracking experimental procedure.

During the experiment, the 24 participants completed the eye-tracking tasks sequentially following a standardized experimental procedure. The experiment consisted of five stages: an introduction page, a gaze calibration page, an instruction page, a stimulus presentation page, and a concluding page (Fig 7). The instruction presented to participants was: “Please carefully observe the following hair dryer color schemes and determine which color scheme you consider to be the optimal one.”

After the experiment, eye-tracking data were exported to generate attention heatmaps (Fig 8). The heatmap results indicate that participants’ visual attention was primarily concentrated on schemes S-06, S-05, and S-04, while relatively less attention was allocated to schemes S-01, S-07, and S-03.

**Fig 8:**
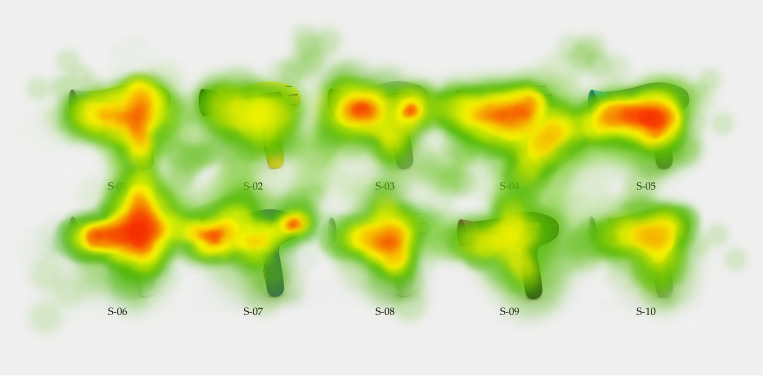
Attention heatmap results.

The average fixation duration for each color scheme was calculated. The original fixation duration statistics for color schemes are provided in S3 File. To facilitate comparison with the evaluation results obtained from the HKES model, the eye-tracking metrics were further normalized, and a ranking of the color schemes was derived, as summarized in Table 8.

**Table 8:** Fixation duration statistics of color schemes.

Scheme	S-01	S-02	S-03	S-04	S-05	S-06	S-07	S-08	S-09	S-10
Mean fixation duration	6.456	3.491	4.502	7.604	7.835	9.148	5.544	4.403	3.360	4.081
Normalized value	0.535	0.023	0.197	0.733	0.773	1.000	0.377	0.180	0.000	0.125
Prioritization	4	9	6	3	2	1	5	7	10	8

The fixation duration results reveal significant differences in visual attention among the ten color schemes. The final ranking based on eye-tracking data is: S-06, S-05, S-04, S-01, S-07, S-03, S-08, S-10, S-02, and S-09. Among them, scheme S-06 exhibits the longest fixation duration, followed by S-05 and S-04, whereas scheme S-09 consistently receives the lowest level of visual attention.

The ranking obtained from eye-tracking data is highly consistent with the evaluation results derived from the HKES model, indicating strong agreement between model-based assessments and users’ actual visual behavior. This consistency demonstrates that the proposed HKES method effectively captures users’ visual preferences in color scheme evaluation. Therefore, the eye-tracking results provide objective empirical evidence supporting the feasibility and effectiveness of the proposed HKES framework.

**Fig 9:**
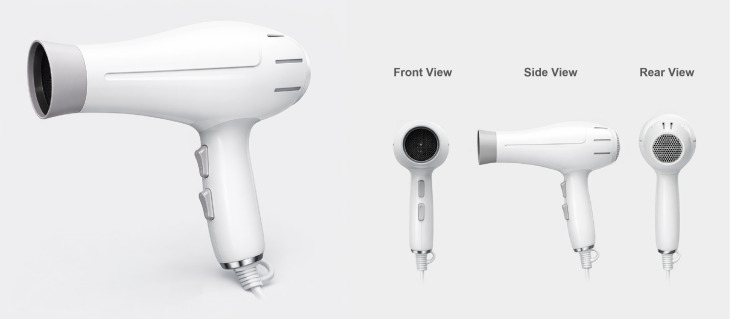
Rendering of the product with the optimal color scheme.

Finally, the color scheme S-06, which achieved the best overall evaluation performance, was selected and applied to the overall design of the hair dryer product. Based on this scheme, further optimization was conducted on the product modeling and rendering, including refinements to surface materials and structural details. The final product design rendering is presented in Fig 9.

## Discussion

This article presents a hybrid product color emotional design method that integrates the PAD emotional model with fuzzy grey relational analysis. It emphasizes a closed-loop process that combines design and evaluation, aiming to reduce reliance on advanced technologies by employing accessible methods for emotional measurement and assessment. Based on hybrid Kansei engineering, the method first establishes a positive design model for analyzing and creating the emotional experience of existing product color schemes. It then constructs a BKES framework to evaluate and prioritize these designs. This approach ensures that, even under limited technical conditions and user familiarity, the design and evaluation of product color schemes can be carried out conveniently and objectively, ultimately enhancing users’ positive emotional experiences with color designs. The application to hair dryers demonstrates its strong feasibility and practicality, offering valuable support for designers.

The evaluation of the emotional experience of hair dryer color design schemes, based on fuzzy grey relational analysis, revealed a priority order: S-06, S-05, S-04, S-01, and S-07. Among these, four are dual-color schemes and one is a tri-color scheme, indicating a preference for dual-color designs. This result is consistent with the findings from the previous PAD scaling measurements.

We can summarize the results as follows: First, Top Design (S-06): Features white as the main color and light gray as the accent, achieving a high fuzzy grey relational degree of 0.955. This scheme aligns well with users’ emotional preferences, highlighting a soft, relaxed aesthetic that complements the clean functionality of hair dryers. Second, Other Preferred Designs (S-05, S-04): These use gray and white with magenta and blue accents, scoring 0.891 and 0.881, respectively. The low saturation and high brightness help avoid negative emotions like harshness or glare. Third, Additional Designs (S-01, S-07): S-01 uses green and gray, while S-07 employs ultramarine, orange, and gray. These designs evoke freshness and vibrancy, appealing to younger consumers seeking trendy aesthetics. Overall, the results indicate that dual-color schemes are generally more suitable for the emotional color design of household hair dryers compared to tricolor combinations.

As this study establishes a hybrid product color emotional design paradigm by integrating the PAD emotional model with fuzzy grey relational analysis, the key aspects of this paradigm include: First, Closed-Loop Process: To address users’ multi-dimensional emotional experiences, the paradigm connects design factor analysis with design scheme evaluation, creating a seamless link between “design” and “evaluation”. Second, Accessible Methods: It reduces reliance on advanced technologies and complex conditions, using manageable and objective methods for color emotional measurement and evaluation. This enables effective design and preference evaluation without specialized equipment. Third, Consistency and Reliability: By using consistent dimensions for both design factors and evaluation, the paradigm enhances the reliability and validity of research outcomes, accurately reflecting user preferences and emotional responses.

Compared with traditional methodologies, this HKES-driven approach extends beyond conventional techniques such as semantic differential analysis and principal component analysis. It takes users’ positive emotional responses to color as the benchmark. By integrating the PAD model with the Chinese PAD emotional scale and Euclidean distance calculations, it conducts a comprehensive analysis of existing color schemes, thereby guiding the development of designs that are more likely to elicit positive emotions. The use of established tools for PAD measurement ensures operational feasibility and reduces common constraints in quantitative analysis, such as environmental limitations and methodological complexity. In particular, during Stage 2, the BKES framework surpasses standard perceptual methods by combining fuzzy membership degree calculations with grey relational analysis, enabling a rational and practical evaluation process. This integrated approach not only identifies optimal color schemes but also provides multiple viable design options, offering flexibility within a defined range.

HKES features bidirectional translation, making it suitable for designing products that meet users’ emotional needs or preferences. In both FKES and BKES, the methodology applies consistent standards when selecting analytical tools and techniques. Specifically, the quantitative analysis of design factors in FKES is based on primary user needs, and vice versa for the emotional or affective evaluation of design schemes in BKES [17,19,20]. This ensures that the selected designs closely match the users’ real-world contexts.

**Fig 10:**
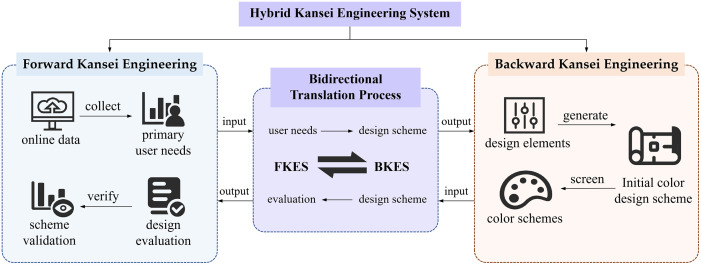
Operational mechanism of the HKES model.

As illustrated in Fig 10, the bidirectional translation mechanism of the proposed HKES model is realized through an explicit interaction between the FKES and BKES. In the FKES process, online data are collected to identify primary user needs, which form the basis for the evaluation and validation of design schemes. The evaluation results provide feedback on how well candidate schemes satisfy users’ emotional needs or preferences. Conversely, in the BKES process, design elements are used to generate initial design schemes, which are subsequently screened and refined according to the evaluation feedback obtained from FKES. Through this iterative bidirectional loop, user needs and design evaluations are continuously translated into design decisions, ensuring consistency between analytical standards and design outcomes.

Product color scheme selection is an affective design problem, as users’ emotional responses to color are subjective and perceptual in nature [63]. This study focuses on the decision-oriented evaluation of a finite set of product color schemes according to their consistency with users’ emotional expectations in the PAD space. Rather than pursuing large-scale affective prediction, the objective is to support design decision-making by enabling systematic comparison and transparent ranking of alternative color schemes within a specific design context. To address this requirement, a HKES paradigm is adopted, as it allows the integration of complementary analytical techniques to jointly consider emotional perception, uncertainty, and comparative evaluation within a unified framework. From a broader methodological perspective, existing KE approaches can be broadly grouped into several representative paradigms. Traditional KE relies primarily on questionnaires and conventional statistical analyses to explore general affective tendencies [64]. HKES extends this foundation by integrating multiple methods for comprehensive design evaluation. Qualitative-analysis-based HKES emphasizes interviews, expert judgment, and thematic analysis, and is mainly applied in early-stage concept exploration. Deep-learning-based HKES focuses on modeling complex non-linear affective relationships from large-scale data. In contrast, the PAD–FGRA–based HKES framework proposed in this study targets decision-oriented evaluation and ranking of design schemes within an explicit emotional space [65], as shown in Table 9.

**Table 9:** Method-level comparison of representative Kansei Engineering paradigms.

Method paradigm	Primary analytical focus	Data requirement	Interpretability	Implementation cost	Application target
Traditional Kansei Engineering	Statistical relationship analysis	Medium	Medium	Medium	General affective trend analysis
Hybrid Kansei Engineering	Multi-method integration	Medium	Medium	Medium	Broad design evaluation problems
Qualitative-analysis-based Hybrid KE	Exploratory affective understanding	Low–Medium	High	Medium	Early-stage concept exploration
Deep-learning-based Hybrid KE	Non-linear affective prediction	High	Low	High	Large-scale affective prediction
PAD–fuzzy GRA–based Hybrid KE (Proposed)	Decision-oriented evaluation and ranking	Low–Medium	High	Low	Color scheme evaluation and selection in PAD space

Among these paradigms, deep-learning-based HKE represents an important methodological trend. By leveraging neural networks, such approaches can effectively model complex non-linear mappings between design features and affective responses, and they are particularly suitable for large-scale affective prediction when sufficient labeled data are available [66]. However, these methods typically involve high data and training requirements, and their limited interpretability may constrain their applicability in design decision-making contexts where transparent evaluation logic and explainable ranking outcomes are required.

The PAD–FGRA framework is adopted in this study because it is well aligned with the characteristics of color emotional design evaluation. First, the evaluation task is inherently comparative, as the objective is to rank a finite number of color schemes based on their relative consistency with users’ emotional expectations. GRA is specifically designed to measure relative closeness among alternatives, making it suitable for scheme ranking. Second, emotional modeling is conducted in the PAD space, which provides a low-dimensional and semantically explicit representation of affective perception. Under such conditions, the analytical focus lies in assessing overall emotional consistency across multiple dimensions rather than learning latent features. GRA provides an effective relational mechanism for aggregating multi-dimensional PAD information. Third, emotional evaluations in design research are often uncertain and subjective, and sample sizes are typically limited. By integrating fuzzy modeling with GRA, the proposed framework can account for uncertainty while maintaining robustness under small-to-moderate sample conditions. Therefore, the adoption of GRA in this study reflects a methodologically appropriate choice driven by the research objective, data characteristics, and application target.

**Fig 11:**
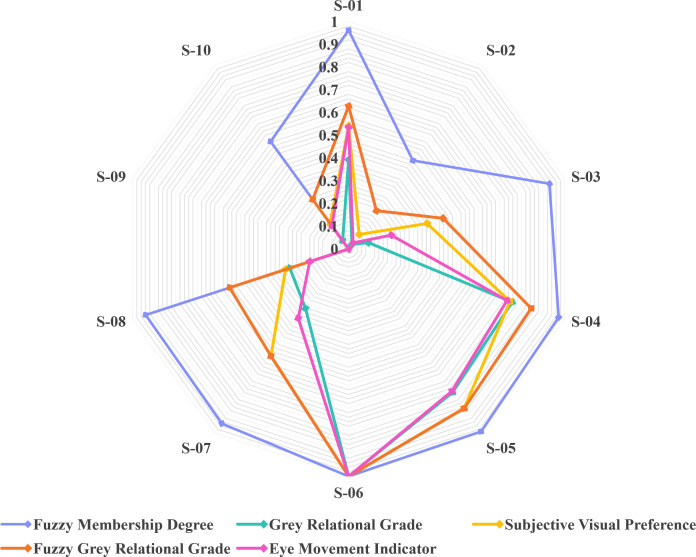
Radar chart comparison of color scheme evaluation results obtained using different methods.

To further complement the above methodological comparison, a data-level comparison was conducted using the same evaluation dataset. Specifically, the proposed PAD-based FGRA method was compared with traditional grey relational analysis without fuzzy modeling, as well as a fuzzy membership degree–based evaluation approach. All methods were applied to the same set of color schemes and PAD-based emotional evaluation data to ensure comparability. As eye-tracking reflects users’ actual visual attention behavior during the perception of design schemes, evaluation results that are more consistent with eye-tracking patterns can be regarded as being closer to users’ real visual cognitive characteristics. Therefore, eye-tracking results were used as a behavioral reference, and a radar chart (Fig 11) was constructed to intuitively compare the evaluation outcomes obtained using different methods across all color schemes. To ensure scientific comparability, the final results for each scheme were normalized prior to visualization.

The five methods exhibit similar overall ranking tendencies, indicating that the proposed framework preserves the fundamental evaluation trend. However, clear differences can be observed in their ability to discriminate among intermediate schemes. The fuzzy membership degree–based results tend to produce relatively concentrated value distributions, with generally high scores. Schemes such as S-01, S-03, S-04, S-05, S-06, S-07, and S-08 cluster near the outer region of the radar chart, showing limited differentiation and thereby restricting discriminative capability. Traditional grey relational analysis improves relative differentiation by introducing relational structure; however, it does not explicitly address the inherent uncertainty and fuzziness in affective evaluation. As a result, the stability of evaluation outcomes for intermediate schemes (e.g., S-01, S-03, and S-07) is relatively weak, and their consistency with PAD-based affective scores and eye-tracking patterns is limited. In contrast, the proposed FGRA method produces smoother and more stable value distributions for mid- and high-ranked color schemes, reflecting the combined effects of fuzzy modeling and relational analysis. More importantly, FGRA-based results demonstrate stronger alignment with both PAD-based affective scores and independent eye-tracking outcomes.

This advantage becomes more evident when examining specific numerical cases. For example, in the mid-to-high ranking region (S-04 to S-07), the traditional grey relational grade drops sharply from 1.000 (S-06) to 0.323 (S-07), whereas the corresponding decrease in the FGRA results is much more gradual, from 1.000 to 0.582. Notably, this FGRA variation closely matches the change observed in PAD-based affective scores (from 1.000 to 0.581), indicating improved structural consistency. Similar patterns can be observed for intermediate schemes such as S-01 and S-03. Traditional grey relational analysis yields relatively low values for these schemes (0.392 and 0.091, respectively), whereas FGRA produces more moderate evaluations (0.628 and 0.437), which are closer to both PAD scores and eye-tracking indicators (0.541/0.535 for S-01 and 0.363/0.197 for S-03). The observed consistency among FGRA, PAD, and eye-tracking results indicates that the proposed framework more effectively captures users’ emotional perception and visual preference structure. Overall, this data-level comparison and behavioral validation provide empirical support for the effectiveness and cognitive plausibility of the proposed hybrid Kansei Engineering framework in decision-oriented color scheme evaluation.

Theoretically, this study extends the hybrid Kansei Engineering framework by establishing a clear closed-loop mechanism that links forward design generation with backward evaluation, thereby enhancing the methodological completeness of emotional design research. Practically, the proposed approach provides a lightweight yet systematic tool for designers, particularly in small appliance industries, to align product color schemes with users’ emotional expectations. The dual emphasis on methodological rigor and practical usability highlights both the academic and industrial value of this work.

## Conclusions and limitations

This study addresses the long-standing challenge of translating users’ complex and often ambiguous emotional responses to product colors into practical and optimized design schemes. By integrating the PAD model with fuzzy grey relational analysis within a hybrid Kansei Engineering framework, it establishes a closed-loop approach that connects emotional measurement with design evaluation, thereby ensuring that user-centered insights can directly inform color design practice. In response to these research questions, several conclusions can be drawn. First, regarding the accurate capture of emotional responses, this study demonstrates that the PAD emotional scale—particularly its localized adaptation—offers a practical and effective tool for quantifying users’ affective experiences with product colors, balancing methodological simplicity with robustness. Second, the establishment of a hybrid Kansei Engineering framework that integrates both forward and backward systems effectively bridges the gap between design and evaluation. The forward system translates emotional responses into color design schemes, while the backward system, supported by fuzzy grey relational analysis, provides systematic evaluation and optimization, thereby ensuring a continuous feedback mechanism. Third, the integration of fuzzy set theory with grey relational analysis enables a rational transformation of complex, multi-dimensional emotional data into manageable evaluation results. This approach makes it possible to rank and prioritize schemes objectively, ultimately identifying those color designs that resonate most strongly with users’ positive emotions. Together, these findings not only confirm the feasibility of the proposed framework but also establish a replicable pathway for emotion-driven product color design.

Clearly, there were several certain shortcomings in our study: The scaling measurements did not account for variables such as income levels, education, or cultural differences in emotional responses. Moreover, the assessments following the measurements did not consider traditional factors like gender and relationship status.

We did acknowledge that variables such as income levels and gender can significantly influence measurements and assessments. However, ensuring the protection of participants’ privacy and related information is essential [67]. In addition, we aim to develop a more diverse and comprehensive emotional experience approach that is de-gendered, or even de-contextualized [68]. Future research will explore these possibilities to enhance the robustness and inclusivity of the methodology.

Product color is undoubtedly a critical factor in consumer decision-making, as it captures users’ visual preferences and emotional needs. This enhances user satisfaction and significantly boosts product competitiveness. In today’s context of addressing users’ emotional needs, design schemes that evoke visual and emotional experiences—beyond color matching—should be carefully designed to align with the aesthetic preferences of the target group while fostering emotional resonance (Barata et al. 2023; Wegman and Said 2011).

This study underscores the pivotal role of product color in consumer decision-making and highlights the importance of aligning design schemes with the aesthetic preferences of target groups to enhance user satisfaction and market competitiveness. Future research will expand participant diversity and increase case samples to better understand how different user groups perceive color-related emotional experiences. It will also explore how variables such as color proportions, shapes, and materials affect emotions across various product types, as well as the influence of factors like gender, occupation, and culture on emotional satisfaction. Furthermore, the study will examine short-term predictions of color design styles for identifying optimal schemes, with the aim of enriching and refining the hybrid emotional design methodology.

## Supporting information

S1 File Color scheme emotional experience questionnaire. (PDF)

S2 File User preference assessment of color-matching schemes. (PDF)

S3 File Fixation duration statistics for color-matching schemes. (PDF)
