# Supplementary material for: A hybrid color emotional experience approach: Integrating the pleasure-arousal-dominance model with fuzzy grey relational analysis
Source: PLoS One. 2026 Feb 2;21(2):e0341895. doi: 10.1371/journal.pone.0341895 (PMC12863556; doi:10.1371/journal.pone.0341895)
Supplement: S1 File — (PDF) [file pone.0341895.s001.pdf]

Color-Matching Scheme Emotional Experience Questionnaire

We are a research team in university, and we are currently conducting a study on emotional experiences induced by different color schemes.This questionnaire aims to assess the emotional experiences elicited by different color schemes, in order to support the analysis and validation of the color design method proposed in this study. The questionnaire is based on the PAD emotional model and evaluates each color scheme along three dimensions: Pleasure, Arousal, and Dominance.

During the evaluation, you will be presented with a series of color scheme samples. For each scheme, please rate the intensity of your emotional experience on the above three dimensions according to your immediate, intuitive impression. Please use the rating scale provided below each item and select the level that best reflects your genuine feelings. There are no “right” or “wrong” answers; we are only interested in your personal impressions.

This questionnaire is for academic research purposes only. All data will be collected and analyzed anonymously and will not involve any personally identifying information. Thank you very much for your participation and cooperation.

**Cases No. <u>C-01</u>**	**PAD Scales**			**Results**
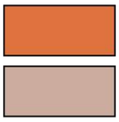	Pleasure, P	Angry-Interested	○-4 ○-3 ○-2 ○-1 ○0 ○1 ○2 ○3 ○4	( )
		Friendly-Dismissive	○-4 ○-3 ○-2 ○-1 ○0 ○1 ○2 ○3 ○4	( )
		Miserable-Happy	○-4 ○-3 ○-2 ○-1 ○0 ○1 ○2 ○3 ○4	( )
		Excited-Irritated	○-4 ○-3 ○-2 ○-1 ○0 ○1 ○2 ○3 ○4	( )
	Arousal, A	Awake-Sleepy	○-4 ○-3 ○-2 ○-1 ○0 ○1 ○2 ○3 ○4	( )
		Calm-Excited	○-4 ○-3 ○-2 ○-1 ○0 ○1 ○2 ○3 ○4	( )
		Interested-Relaxed	○-4 ○-3 ○-2 ○-1 ○0 ○1 ○2 ○3 ○4	( )
		Stiff-Surprised	○-4 ○-3 ○-2 ○-1 ○0 ○1 ○2 ○3 ○4	( )
	Dominance, D	Controlled-Master	○-4 ○-3 ○-2 ○-1 ○0 ○1 ○2 ○3 ○4	( )
		Dominant-Submissive	○-4 ○-3 ○-2 ○-1 ○0 ○1 ○2 ○3 ○4	( )
		Humble-Arrogant	○-4 ○-3 ○-2 ○-1 ○0 ○1 ○2 ○3 ○4	( )
		Influential-Affected	○-4 ○-3 ○-2 ○-1 ○0 ○1 ○2 ○3 ○4	( )
**Cases No. <u>C-02</u>**	**PAD Scales**			**Results**
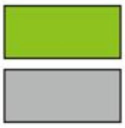	Pleasure, P	Angry-Interested	○-4 ○-3 ○-2 ○-1 ○0 ○1 ○2 ○3 ○4	( )
		Friendly-Dismissive	○-4 ○-3 ○-2 ○-1 ○0 ○1 ○2 ○3 ○4	( )
		Miserable-Happy	○-4 ○-3 ○-2 ○-1 ○0 ○1 ○2 ○3 ○4	( )
		Excited-Irritated	○-4 ○-3 ○-2 ○-1 ○0 ○1 ○2 ○3 ○4	( )
	Arousal,	Awake-Sleepy	○-4 ○-3 ○-2 ○-1 ○0 ○1 ○2 ○3 ○4	( )

	A	Calm-Excited	○-4 ○-3 ○-2 ○-1 ○0 ○1 ○2 ○3 ○4	( )
		Interested-Relaxed	○-4 ○-3 ○-2 ○-1 ○0 ○1 ○2 ○3 ○4	( )
		Stiff-Surprised	○-4 ○-3 ○-2 ○-1 ○0 ○1 ○2 ○3 ○4	( )
	Dominance, D	Controlled-Master	○-4 ○-3 ○-2 ○-1 ○0 ○1 ○2 ○3 ○4	( )
		Dominant-Submissive	○-4 ○-3 ○-2 ○-1 ○0 ○1 ○2 ○3 ○4	( )
		Humble-Arrogant	○-4 ○-3 ○-2 ○-1 ○0 ○1 ○2 ○3 ○4	( )
		Influential-Affected	○-4 ○-3 ○-2 ○-1 ○0 ○1 ○2 ○3 ○4	( )
**Cases No. <u>C-03</u>**	**PAD Scales**			**Results**
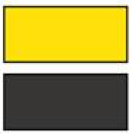	Pleasure, P	Angry-Interested	○-4 ○-3 ○-2 ○-1 ○0 ○1 ○2 ○3 ○4	( )
		Friendly-Dismissive	○-4 ○-3 ○-2 ○-1 ○0 ○1 ○2 ○3 ○4	( )
		Miserable-Happy	○-4 ○-3 ○-2 ○-1 ○0 ○1 ○2 ○3 ○4	( )
		Excited-Irritated	○-4 ○-3 ○-2 ○-1 ○0 ○1 ○2 ○3 ○4	( )
	Arousal, A	Awake-Sleepy	○-4 ○-3 ○-2 ○-1 ○0 ○1 ○2 ○3 ○4	( )
		Calm-Excited	○-4 ○-3 ○-2 ○-1 ○0 ○1 ○2 ○3 ○4	( )
		Interested-Relaxed	○-4 ○-3 ○-2 ○-1 ○0 ○1 ○2 ○3 ○4	( )
		Stiff-Surprised	○-4 ○-3 ○-2 ○-1 ○0 ○1 ○2 ○3 ○4	( )
	Dominance, D	Controlled-Master	○-4 ○-3 ○-2 ○-1 ○0 ○1 ○2 ○3 ○4	( )
		Dominant-Submissive	○-4 ○-3 ○-2 ○-1 ○0 ○1 ○2 ○3 ○4	( )
		Humble-Arrogant	○-4 ○-3 ○-2 ○-1 ○0 ○1 ○2 ○3 ○4	( )
		Influential-Affected	○-4 ○-3 ○-2 ○-1 ○0 ○1 ○2 ○3 ○4	( )
**Cases No. <u>C-04</u>**	**PAD Scales**			**Results**
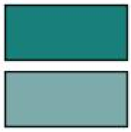	Pleasure, P	Angry-Interested	○-4 ○-3 ○-2 ○-1 ○0 ○1 ○2 ○3 ○4	( )
		Friendly-Dismissive	○-4 ○-3 ○-2 ○-1 ○0 ○1 ○2 ○3 ○4	( )
		Miserable-Happy	○-4 ○-3 ○-2 ○-1 ○0 ○1 ○2 ○3 ○4	( )
		Excited-Irritated	○-4 ○-3 ○-2 ○-1 ○0 ○1 ○2 ○3 ○4	( )
	Arousal, A	Awake-Sleepy	○-4 ○-3 ○-2 ○-1 ○0 ○1 ○2 ○3 ○4	( )
		Calm-Excited	○-4 ○-3 ○-2 ○-1 ○0 ○1 ○2 ○3 ○4	( )
		Interested-Relaxed	○-4 ○-3 ○-2 ○-1 ○0 ○1 ○2 ○3 ○4	( )
		Stiff-Surprised	○-4 ○-3 ○-2 ○-1 ○0 ○1 ○2 ○3 ○4	( )
	Dominance, D	Controlled-Master	○-4 ○-3 ○-2 ○-1 ○0 ○1 ○2 ○3 ○4	( )
		Dominant-Submissive	○-4 ○-3 ○-2 ○-1 ○0 ○1 ○2 ○3 ○4	( )
		Humble-Arrogant	○-4 ○-3 ○-2 ○-1 ○0 ○1 ○2 ○3 ○4	( )
		Influential-Affected	○-4 ○-3 ○-2 ○-1 ○0 ○1 ○2 ○3 ○4	( )
**Cases No. <u>C-05</u>**	**PAD Scales**			**Results**
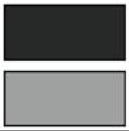	Pleasure, P	Angry-Interested	○-4 ○-3 ○-2 ○-1 ○0 ○1 ○2 ○3 ○4	( )
		Friendly-Dismissive	○-4 ○-3 ○-2 ○-1 ○0 ○1 ○2 ○3 ○4	( )
		Miserable-Happy	○-4 ○-3 ○-2 ○-1 ○0 ○1 ○2 ○3 ○4	( )
		Excited-Irritated	○-4 ○-3 ○-2 ○-1 ○0 ○1 ○2 ○3 ○4	( )
	Arousal,	Awake-Sleepy	○-4 ○-3 ○-2 ○-1 ○0 ○1 ○2 ○3 ○4	( )

	A	Calm-Excited	○-4 ○-3 ○-2 ○-1 ○0 ○1 ○2 ○3 ○4	( )
		Interested-Relaxed	○-4 ○-3 ○-2 ○-1 ○0 ○1 ○2 ○3 ○4	( )
		Stiff-Surprised	○-4 ○-3 ○-2 ○-1 ○0 ○1 ○2 ○3 ○4	( )
	Dominance, D	Controlled-Master	○-4 ○-3 ○-2 ○-1 ○0 ○1 ○2 ○3 ○4	( )
		Dominant-Submissive	○-4 ○-3 ○-2 ○-1 ○0 ○1 ○2 ○3 ○4	( )
		Humble-Arrogant	○-4 ○-3 ○-2 ○-1 ○0 ○1 ○2 ○3 ○4	( )
		Influential-Affected	○-4 ○-3 ○-2 ○-1 ○0 ○1 ○2 ○3 ○4	( )
**Cases No. C-06**	**PAD Scales**			**Results**
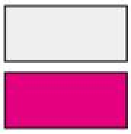	Pleasure, P	Angry-Interested	○-4 ○-3 ○-2 ○-1 ○0 ○1 ○2 ○3 ○4	( )
		Friendly-Dismissive	○-4 ○-3 ○-2 ○-1 ○0 ○1 ○2 ○3 ○4	( )
		Miserable-Happy	○-4 ○-3 ○-2 ○-1 ○0 ○1 ○2 ○3 ○4	( )
		Excited-Irritated	○-4 ○-3 ○-2 ○-1 ○0 ○1 ○2 ○3 ○4	( )
	Arousal, A	Awake-Sleepy	○-4 ○-3 ○-2 ○-1 ○0 ○1 ○2 ○3 ○4	( )
		Calm-Excited	○-4 ○-3 ○-2 ○-1 ○0 ○1 ○2 ○3 ○4	( )
		Interested-Relaxed	○-4 ○-3 ○-2 ○-1 ○0 ○1 ○2 ○3 ○4	( )
		Stiff-Surprised	○-4 ○-3 ○-2 ○-1 ○0 ○1 ○2 ○3 ○4	( )
	Dominance, D	Controlled-Master	○-4 ○-3 ○-2 ○-1 ○0 ○1 ○2 ○3 ○4	( )
		Dominant-Submissive	○-4 ○-3 ○-2 ○-1 ○0 ○1 ○2 ○3 ○4	( )
		Humble-Arrogant	○-4 ○-3 ○-2 ○-1 ○0 ○1 ○2 ○3 ○4	( )
		Influential-Affected	○-4 ○-3 ○-2 ○-1 ○0 ○1 ○2 ○3 ○4	( )
**Cases No. C-07**	**PAD Scales**			**Results**
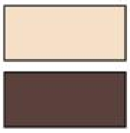	Pleasure, P	Angry-Interested	○-4 ○-3 ○-2 ○-1 ○0 ○1 ○2 ○3 ○4	( )
		Friendly-Dismissive	○-4 ○-3 ○-2 ○-1 ○0 ○1 ○2 ○3 ○4	( )
		Miserable-Happy	○-4 ○-3 ○-2 ○-1 ○0 ○1 ○2 ○3 ○4	( )
		Excited-Irritated	○-4 ○-3 ○-2 ○-1 ○0 ○1 ○2 ○3 ○4	( )
	Arousal, A	Awake-Sleepy	○-4 ○-3 ○-2 ○-1 ○0 ○1 ○2 ○3 ○4	( )
		Calm-Excited	○-4 ○-3 ○-2 ○-1 ○0 ○1 ○2 ○3 ○4	( )
		Interested-Relaxed	○-4 ○-3 ○-2 ○-1 ○0 ○1 ○2 ○3 ○4	( )
		Stiff-Surprised	○-4 ○-3 ○-2 ○-1 ○0 ○1 ○2 ○3 ○4	( )
	Dominance, D	Controlled-Master	○-4 ○-3 ○-2 ○-1 ○0 ○1 ○2 ○3 ○4	( )
		Dominant-Submissive	○-4 ○-3 ○-2 ○-1 ○0 ○1 ○2 ○3 ○4	( )
		Humble-Arrogant	○-4 ○-3 ○-2 ○-1 ○0 ○1 ○2 ○3 ○4	( )
		Influential-Affected	○-4 ○-3 ○-2 ○-1 ○0 ○1 ○2 ○3 ○4	( )
**Cases No. C-08**	**PAD Scales**			**Results**
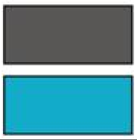	Pleasure, P	Angry-Interested	○-4 ○-3 ○-2 ○-1 ○0 ○1 ○2 ○3 ○4	( )
		Friendly-Dismissive	○-4 ○-3 ○-2 ○-1 ○0 ○1 ○2 ○3 ○4	( )
		Miserable-Happy	○-4 ○-3 ○-2 ○-1 ○0 ○1 ○2 ○3 ○4	( )
		Excited-Irritated	○-4 ○-3 ○-2 ○-1 ○0 ○1 ○2 ○3 ○4	( )
	Arousal,	Awake-Sleepy	○-4 ○-3 ○-2 ○-1 ○0 ○1 ○2 ○3 ○4	( )

	A	Calm-Excited	○-4 ○-3 ○-2 ○-1 ○0 ○1 ○2 ○3 ○4	( )
		Interested-Relaxed	○-4 ○-3 ○-2 ○-1 ○0 ○1 ○2 ○3 ○4	( )
		Stiff-Surprised	○-4 ○-3 ○-2 ○-1 ○0 ○1 ○2 ○3 ○4	( )
	Dominance, D	Controlled-Master	○-4 ○-3 ○-2 ○-1 ○0 ○1 ○2 ○3 ○4	( )
		Dominant-Submissive	○-4 ○-3 ○-2 ○-1 ○0 ○1 ○2 ○3 ○4	( )
		Humble-Arrogant	○-4 ○-3 ○-2 ○-1 ○0 ○1 ○2 ○3 ○4	( )
		Influential-Affected	○-4 ○-3 ○-2 ○-1 ○0 ○1 ○2 ○3 ○4	( )
**Cases No. <u>C-09</u>**	**PAD Scales**			**Results**
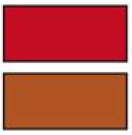	Pleasure, P	Angry-Interested	○-4 ○-3 ○-2 ○-1 ○0 ○1 ○2 ○3 ○4	( )
		Friendly-Dismissive	○-4 ○-3 ○-2 ○-1 ○0 ○1 ○2 ○3 ○4	( )
		Miserable-Happy	○-4 ○-3 ○-2 ○-1 ○0 ○1 ○2 ○3 ○4	( )
		Excited-Irritated	○-4 ○-3 ○-2 ○-1 ○0 ○1 ○2 ○3 ○4	( )
	Arousal, A	Awake-Sleepy	○-4 ○-3 ○-2 ○-1 ○0 ○1 ○2 ○3 ○4	( )
		Calm-Excited	○-4 ○-3 ○-2 ○-1 ○0 ○1 ○2 ○3 ○4	( )
		Interested-Relaxed	○-4 ○-3 ○-2 ○-1 ○0 ○1 ○2 ○3 ○4	( )
		Stiff-Surprised	○-4 ○-3 ○-2 ○-1 ○0 ○1 ○2 ○3 ○4	( )
	Dominance, D	Controlled-Master	○-4 ○-3 ○-2 ○-1 ○0 ○1 ○2 ○3 ○4	( )
		Dominant-Submissive	○-4 ○-3 ○-2 ○-1 ○0 ○1 ○2 ○3 ○4	( )
		Humble-Arrogant	○-4 ○-3 ○-2 ○-1 ○0 ○1 ○2 ○3 ○4	( )
		Influential-Affected	○-4 ○-3 ○-2 ○-1 ○0 ○1 ○2 ○3 ○4	( )
**Cases No. <u>C-10</u>**	**PAD Scales**			**Results**
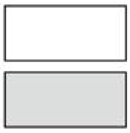	Pleasure, P	Angry-Interested	○-4 ○-3 ○-2 ○-1 ○0 ○1 ○2 ○3 ○4	( )
		Friendly-Dismissive	○-4 ○-3 ○-2 ○-1 ○0 ○1 ○2 ○3 ○4	( )
		Miserable-Happy	○-4 ○-3 ○-2 ○-1 ○0 ○1 ○2 ○3 ○4	( )
		Excited-Irritated	○-4 ○-3 ○-2 ○-1 ○0 ○1 ○2 ○3 ○4	( )
	Arousal, A	Awake-Sleepy	○-4 ○-3 ○-2 ○-1 ○0 ○1 ○2 ○3 ○4	( )
		Calm-Excited	○-4 ○-3 ○-2 ○-1 ○0 ○1 ○2 ○3 ○4	( )
		Interested-Relaxed	○-4 ○-3 ○-2 ○-1 ○0 ○1 ○2 ○3 ○4	( )
		Stiff-Surprised	○-4 ○-3 ○-2 ○-1 ○0 ○1 ○2 ○3 ○4	( )
	Dominance, D	Controlled-Master	○-4 ○-3 ○-2 ○-1 ○0 ○1 ○2 ○3 ○4	( )
		Dominant-Submissive	○-4 ○-3 ○-2 ○-1 ○0 ○1 ○2 ○3 ○4	( )
		Humble-Arrogant	○-4 ○-3 ○-2 ○-1 ○0 ○1 ○2 ○3 ○4	( )
		Influential-Affected	○-4 ○-3 ○-2 ○-1 ○0 ○1 ○2 ○3 ○4	( )
**Cases No. <u>C-11</u>**	**PAD Scales**			**Results**
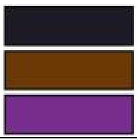	Pleasure, P	Angry-Interested	○-4 ○-3 ○-2 ○-1 ○0 ○1 ○2 ○3 ○4	( )
		Friendly-Dismissive	○-4 ○-3 ○-2 ○-1 ○0 ○1 ○2 ○3 ○4	( )
		Miserable-Happy	○-4 ○-3 ○-2 ○-1 ○0 ○1 ○2 ○3 ○4	( )
		Excited-Irritated	○-4 ○-3 ○-2 ○-1 ○0 ○1 ○2 ○3 ○4	( )
	Arousal,	Awake-Sleepy	○-4 ○-3 ○-2 ○-1 ○0 ○1 ○2 ○3 ○4	( )

	A	Calm-Excited	○-4 ○-3 ○-2 ○-1 ○0 ○1 ○2 ○3 ○4	( )
		Interested-Relaxed	○-4 ○-3 ○-2 ○-1 ○0 ○1 ○2 ○3 ○4	( )
		Stiff-Surprised	○-4 ○-3 ○-2 ○-1 ○0 ○1 ○2 ○3 ○4	( )
	Dominance, D	Controlled-Master	○-4 ○-3 ○-2 ○-1 ○0 ○1 ○2 ○3 ○4	( )
		Dominant-Submissive	○-4 ○-3 ○-2 ○-1 ○0 ○1 ○2 ○3 ○4	( )
		Humble-Arrogant	○-4 ○-3 ○-2 ○-1 ○0 ○1 ○2 ○3 ○4	( )
		Influential-Affected	○-4 ○-3 ○-2 ○-1 ○0 ○1 ○2 ○3 ○4	( )
**Cases No. <u>C-12</u>**	**PAD Scales**			**Results**
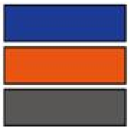	Pleasure, P	Angry-Interested	○-4 ○-3 ○-2 ○-1 ○0 ○1 ○2 ○3 ○4	( )
		Friendly-Dismissive	○-4 ○-3 ○-2 ○-1 ○0 ○1 ○2 ○3 ○4	( )
		Miserable-Happy	○-4 ○-3 ○-2 ○-1 ○0 ○1 ○2 ○3 ○4	( )
		Excited-Irritated	○-4 ○-3 ○-2 ○-1 ○0 ○1 ○2 ○3 ○4	( )
	Arousal, A	Awake-Sleepy	○-4 ○-3 ○-2 ○-1 ○0 ○1 ○2 ○3 ○4	( )
		Calm-Excited	○-4 ○-3 ○-2 ○-1 ○0 ○1 ○2 ○3 ○4	( )
		Interested-Relaxed	○-4 ○-3 ○-2 ○-1 ○0 ○1 ○2 ○3 ○4	( )
		Stiff-Surprised	○-4 ○-3 ○-2 ○-1 ○0 ○1 ○2 ○3 ○4	( )
	Dominance, D	Controlled-Master	○-4 ○-3 ○-2 ○-1 ○0 ○1 ○2 ○3 ○4	( )
		Dominant-Submissive	○-4 ○-3 ○-2 ○-1 ○0 ○1 ○2 ○3 ○4	( )
		Humble-Arrogant	○-4 ○-3 ○-2 ○-1 ○0 ○1 ○2 ○3 ○4	( )
		Influential-Affected	○-4 ○-3 ○-2 ○-1 ○0 ○1 ○2 ○3 ○4	( )
**Cases No. <u>C-13</u>**	**PAD Scales**			**Results**
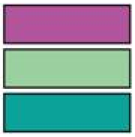	Pleasure, P	Angry-Interested	○-4 ○-3 ○-2 ○-1 ○0 ○1 ○2 ○3 ○4	( )
		Friendly-Dismissive	○-4 ○-3 ○-2 ○-1 ○0 ○1 ○2 ○3 ○4	( )
		Miserable-Happy	○-4 ○-3 ○-2 ○-1 ○0 ○1 ○2 ○3 ○4	( )
		Excited-Irritated	○-4 ○-3 ○-2 ○-1 ○0 ○1 ○2 ○3 ○4	( )
	Arousal, A	Awake-Sleepy	○-4 ○-3 ○-2 ○-1 ○0 ○1 ○2 ○3 ○4	( )
		Calm-Excited	○-4 ○-3 ○-2 ○-1 ○0 ○1 ○2 ○3 ○4	( )
		Interested-Relaxed	○-4 ○-3 ○-2 ○-1 ○0 ○1 ○2 ○3 ○4	( )
		Stiff-Surprised	○-4 ○-3 ○-2 ○-1 ○0 ○1 ○2 ○3 ○4	( )
	Dominance, D	Controlled-Master	○-4 ○-3 ○-2 ○-1 ○0 ○1 ○2 ○3 ○4	( )
		Dominant-Submissive	○-4 ○-3 ○-2 ○-1 ○0 ○1 ○2 ○3 ○4	( )
		Humble-Arrogant	○-4 ○-3 ○-2 ○-1 ○0 ○1 ○2 ○3 ○4	( )
		Influential-Affected	○-4 ○-3 ○-2 ○-1 ○0 ○1 ○2 ○3 ○4	( )
**Cases No. <u>C-14</u>**	**PAD Scales**			**Results**
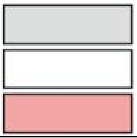	Pleasure, P	Angry-Interested	○-4 ○-3 ○-2 ○-1 ○0 ○1 ○2 ○3 ○4	( )
		Friendly-Dismissive	○-4 ○-3 ○-2 ○-1 ○0 ○1 ○2 ○3 ○4	( )
		Miserable-Happy	○-4 ○-3 ○-2 ○-1 ○0 ○1 ○2 ○3 ○4	( )
		Excited-Irritated	○-4 ○-3 ○-2 ○-1 ○0 ○1 ○2 ○3 ○4	( )
	Arousal,	Awake-Sleepy	○-4 ○-3 ○-2 ○-1 ○0 ○1 ○2 ○3 ○4	( )

	A	Calm-Excited	○-4 ○-3 ○-2 ○-1 ○0 ○1 ○2 ○3 ○4	( )
		Interested-Relaxed	○-4 ○-3 ○-2 ○-1 ○0 ○1 ○2 ○3 ○4	( )
		Stiff-Surprised	○-4 ○-3 ○-2 ○-1 ○0 ○1 ○2 ○3 ○4	( )
	Dominance, D	Controlled-Master	○-4 ○-3 ○-2 ○-1 ○0 ○1 ○2 ○3 ○4	( )
		Dominant-Submissive	○-4 ○-3 ○-2 ○-1 ○0 ○1 ○2 ○3 ○4	( )
		Humble-Arrogant	○-4 ○-3 ○-2 ○-1 ○0 ○1 ○2 ○3 ○4	( )
		Influential-Affected	○-4 ○-3 ○-2 ○-1 ○0 ○1 ○2 ○3 ○4	( )
**Cases No. <u>C-15</u>**	**PAD Scales**			**Results**
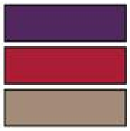	Pleasure, P	Angry-Interested	○-4 ○-3 ○-2 ○-1 ○0 ○1 ○2 ○3 ○4	( )
		Friendly-Dismissive	○-4 ○-3 ○-2 ○-1 ○0 ○1 ○2 ○3 ○4	( )
		Miserable-Happy	○-4 ○-3 ○-2 ○-1 ○0 ○1 ○2 ○3 ○4	( )
		Excited-Irritated	○-4 ○-3 ○-2 ○-1 ○0 ○1 ○2 ○3 ○4	( )
	Arousal, A	Awake-Sleepy	○-4 ○-3 ○-2 ○-1 ○0 ○1 ○2 ○3 ○4	( )
		Calm-Excited	○-4 ○-3 ○-2 ○-1 ○0 ○1 ○2 ○3 ○4	( )
		Interested-Relaxed	○-4 ○-3 ○-2 ○-1 ○0 ○1 ○2 ○3 ○4	( )
		Stiff-Surprised	○-4 ○-3 ○-2 ○-1 ○0 ○1 ○2 ○3 ○4	( )
	Dominance, D	Controlled-Master	○-4 ○-3 ○-2 ○-1 ○0 ○1 ○2 ○3 ○4	( )
		Dominant-Submissive	○-4 ○-3 ○-2 ○-1 ○0 ○1 ○2 ○3 ○4	( )
		Humble-Arrogant	○-4 ○-3 ○-2 ○-1 ○0 ○1 ○2 ○3 ○4	( )
		Influential-Affected	○-4 ○-3 ○-2 ○-1 ○0 ○1 ○2 ○3 ○4	( )
**Cases No. <u>C-16</u>**	**PAD Scales**			**Results**
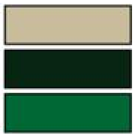	Pleasure, P	Angry-Interested	○-4 ○-3 ○-2 ○-1 ○0 ○1 ○2 ○3 ○4	( )
		Friendly-Dismissive	○-4 ○-3 ○-2 ○-1 ○0 ○1 ○2 ○3 ○4	( )
		Miserable-Happy	○-4 ○-3 ○-2 ○-1 ○0 ○1 ○2 ○3 ○4	( )
		Excited-Irritated	○-4 ○-3 ○-2 ○-1 ○0 ○1 ○2 ○3 ○4	( )
	Arousal, A	Awake-Sleepy	○-4 ○-3 ○-2 ○-1 ○0 ○1 ○2 ○3 ○4	( )
		Calm-Excited	○-4 ○-3 ○-2 ○-1 ○0 ○1 ○2 ○3 ○4	( )
		Interested-Relaxed	○-4 ○-3 ○-2 ○-1 ○0 ○1 ○2 ○3 ○4	( )
		Stiff-Surprised	○-4 ○-3 ○-2 ○-1 ○0 ○1 ○2 ○3 ○4	( )
	Dominance, D	Controlled-Master	○-4 ○-3 ○-2 ○-1 ○0 ○1 ○2 ○3 ○4	( )
		Dominant-Submissive	○-4 ○-3 ○-2 ○-1 ○0 ○1 ○2 ○3 ○4	( )
		Humble-Arrogant	○-4 ○-3 ○-2 ○-1 ○0 ○1 ○2 ○3 ○4	( )
		Influential-Affected	○-4 ○-3 ○-2 ○-1 ○0 ○1 ○2 ○3 ○4	( )
**Cases No. <u>C-17</u>**	**PAD Scales**			**Results**
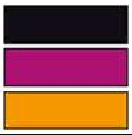	Pleasure, P	Angry-Interested	○-4 ○-3 ○-2 ○-1 ○0 ○1 ○2 ○3 ○4	( )
		Friendly-Dismissive	○-4 ○-3 ○-2 ○-1 ○0 ○1 ○2 ○3 ○4	( )
		Miserable-Happy	○-4 ○-3 ○-2 ○-1 ○0 ○1 ○2 ○3 ○4	( )
		Excited-Irritated	○-4 ○-3 ○-2 ○-1 ○0 ○1 ○2 ○3 ○4	( )
	Arousal,	Awake-Sleepy	○-4 ○-3 ○-2 ○-1 ○0 ○1 ○2 ○3 ○4	( )

	A	Calm-Excited	○-4 ○-3 ○-2 ○-1 ○0 ○1 ○2 ○3 ○4	( )
		Interested-Relaxed	○-4 ○-3 ○-2 ○-1 ○0 ○1 ○2 ○3 ○4	( )
		Stiff-Surprised	○-4 ○-3 ○-2 ○-1 ○0 ○1 ○2 ○3 ○4	( )
	Dominance, D	Controlled-Master	○-4 ○-3 ○-2 ○-1 ○0 ○1 ○2 ○3 ○4	( )
		Dominant-Submissive	○-4 ○-3 ○-2 ○-1 ○0 ○1 ○2 ○3 ○4	( )
		Humble-Arrogant	○-4 ○-3 ○-2 ○-1 ○0 ○1 ○2 ○3 ○4	( )
		Influential-Affected	○-4 ○-3 ○-2 ○-1 ○0 ○1 ○2 ○3 ○4	( )
**Cases No. <u>C-18</u>**	**PAD Scales**			**Results**
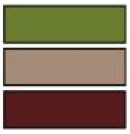	Pleasure, P	Angry-Interested	○-4 ○-3 ○-2 ○-1 ○0 ○1 ○2 ○3 ○4	( )
		Friendly-Dismissive	○-4 ○-3 ○-2 ○-1 ○0 ○1 ○2 ○3 ○4	( )
		Miserable-Happy	○-4 ○-3 ○-2 ○-1 ○0 ○1 ○2 ○3 ○4	( )
		Excited-Irritated	○-4 ○-3 ○-2 ○-1 ○0 ○1 ○2 ○3 ○4	( )
	Arousal, A	Awake-Sleepy	○-4 ○-3 ○-2 ○-1 ○0 ○1 ○2 ○3 ○4	( )
		Calm-Excited	○-4 ○-3 ○-2 ○-1 ○0 ○1 ○2 ○3 ○4	( )
		Interested-Relaxed	○-4 ○-3 ○-2 ○-1 ○0 ○1 ○2 ○3 ○4	( )
		Stiff-Surprised	○-4 ○-3 ○-2 ○-1 ○0 ○1 ○2 ○3 ○4	( )
	Dominance, D	Controlled-Master	○-4 ○-3 ○-2 ○-1 ○0 ○1 ○2 ○3 ○4	( )
		Dominant-Submissive	○-4 ○-3 ○-2 ○-1 ○0 ○1 ○2 ○3 ○4	( )
		Humble-Arrogant	○-4 ○-3 ○-2 ○-1 ○0 ○1 ○2 ○3 ○4	( )
		Influential-Affected	○-4 ○-3 ○-2 ○-1 ○0 ○1 ○2 ○3 ○4	( )
**Cases No. <u>C-19</u>**	**PAD Scales**			**Results**
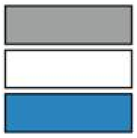	Pleasure, P	Angry-Interested	○-4 ○-3 ○-2 ○-1 ○0 ○1 ○2 ○3 ○4	( )
		Friendly-Dismissive	○-4 ○-3 ○-2 ○-1 ○0 ○1 ○2 ○3 ○4	( )
		Miserable-Happy	○-4 ○-3 ○-2 ○-1 ○0 ○1 ○2 ○3 ○4	( )
		Excited-Irritated	○-4 ○-3 ○-2 ○-1 ○0 ○1 ○2 ○3 ○4	( )
	Arousal, A	Awake-Sleepy	○-4 ○-3 ○-2 ○-1 ○0 ○1 ○2 ○3 ○4	( )
		Calm-Excited	○-4 ○-3 ○-2 ○-1 ○0 ○1 ○2 ○3 ○4	( )
		Interested-Relaxed	○-4 ○-3 ○-2 ○-1 ○0 ○1 ○2 ○3 ○4	( )
		Stiff-Surprised	○-4 ○-3 ○-2 ○-1 ○0 ○1 ○2 ○3 ○4	( )
	Dominance, D	Controlled-Master	○-4 ○-3 ○-2 ○-1 ○0 ○1 ○2 ○3 ○4	( )
		Dominant-Submissive	○-4 ○-3 ○-2 ○-1 ○0 ○1 ○2 ○3 ○4	( )
		Humble-Arrogant	○-4 ○-3 ○-2 ○-1 ○0 ○1 ○2 ○3 ○4	( )
		Influential-Affected	○-4 ○-3 ○-2 ○-1 ○0 ○1 ○2 ○3 ○4	( )
**Cases No. <u>C-20</u>**	**PAD Scales**			**Results**
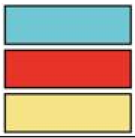	Pleasure, P	Angry-Interested	○-4 ○-3 ○-2 ○-1 ○0 ○1 ○2 ○3 ○4	( )
		Friendly-Dismissive	○-4 ○-3 ○-2 ○-1 ○0 ○1 ○2 ○3 ○4	( )
		Miserable-Happy	○-4 ○-3 ○-2 ○-1 ○0 ○1 ○2 ○3 ○4	( )
		Excited-Irritated	○-4 ○-3 ○-2 ○-1 ○0 ○1 ○2 ○3 ○4	( )
	Arousal,	Awake-Sleepy	○-4 ○-3 ○-2 ○-1 ○0 ○1 ○2 ○3 ○4	( )

	A	Calm-Excited	○-4 ○-3 ○-2 ○-1 ○0 ○1 ○2 ○3 ○4	( )
		Interested-Relaxed	○-4 ○-3 ○-2 ○-1 ○0 ○1 ○2 ○3 ○4	( )
		Stiff-Surprised	○-4 ○-3 ○-2 ○-1 ○0 ○1 ○2 ○3 ○4	( )
	Dominance, D	Controlled-Master	○-4 ○-3 ○-2 ○-1 ○0 ○1 ○2 ○3 ○4	( )
		Dominant-Submissive	○-4 ○-3 ○-2 ○-1 ○0 ○1 ○2 ○3 ○4	( )
		Humble-Arrogant	○-4 ○-3 ○-2 ○-1 ○0 ○1 ○2 ○3 ○4	( )
		Influential-Affected	○-4 ○-3 ○-2 ○-1 ○0 ○1 ○2 ○3 ○4	( )
