# Supplementary material for: A hybrid color emotional experience approach: Integrating the pleasure-arousal-dominance model with fuzzy grey relational analysis
Source: PLoS One. 2026 Feb 2;21(2):e0341895. doi: 10.1371/journal.pone.0341895 (PMC12863556; doi:10.1371/journal.pone.0341895)
Supplement: S2 File — (PDF) [file pone.0341895.s002.pdf]

User Preference Assessment of Color-Matching Schemes

we are a research team from XXX University. This questionnaire is designed to evaluate your emotional responses and subjective preference for different color-matching schemes. The evaluation is based on the PAD emotion model and includes four indexes: Pleasure (P), Arousal (A), Dominance (D), and overall Satisfaction (S).

You will be presented with several color-matching schemes (e.g., S-01, S-02, etc.). For each scheme, please rate your feelings on the four indexes using a nine-point bipolar scale ranging from −4 to +4, where −4 indicates a very negative feeling, +4 indicates a very positive feeling, and 0 represents a neutral feeling. Please select one option for each index according to your intuitive overall impression. There are no “right” or “wrong” answers; we are only interested in your genuine personal evaluation.

This questionnaire is used solely for academic research. All responses will be collected and analyzed anonymously and will not involve any personal identifying information. Thank you very much for your participation and cooperation.

**Schemes No. <u>S-01</u>**	**Evaluation Indexes**	**Evaluation Value**	**Results**
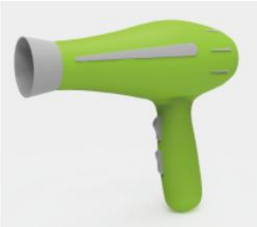	Pleasure, P	○-4 ○-3 ○-2 ○-1 ○0 ○1 ○2 ○3 ○4	( )
	Arousal, A	○-4 ○-3 ○-2 ○-1 ○0 ○1 ○2 ○3 ○4	( )
	Dominance, D	○-4 ○-3 ○-2 ○-1 ○0 ○1 ○2 ○3 ○4	( )
	Satisfaction, S	○-4 ○-3 ○-2 ○-1 ○0 ○1 ○2 ○3 ○4	( )
**Schemes No. <u>S-02</u>**	**Evaluation Indexes**	**Evaluation Value**	**Results**
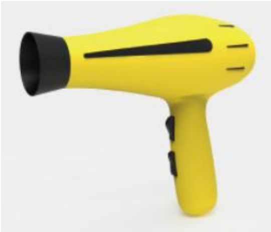	Pleasure, P	○-4 ○-3 ○-2 ○-1 ○0 ○1 ○2 ○3 ○4	( )
	Arousal, A	○-4 ○-3 ○-2 ○-1 ○0 ○1 ○2 ○3 ○4	( )
	Dominance, D	○-4 ○-3 ○-2 ○-1 ○0 ○1 ○2 ○3 ○4	( )
	Satisfaction, S	○-4 ○-3 ○-2 ○-1 ○0 ○1 ○2 ○3 ○4	( )
**Schemes No. <u>S-03</u>**	**Evaluation Indexes**	**Evaluation Value**	**Results**
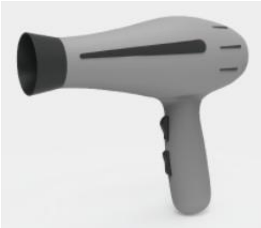	Pleasure, P	○-4 ○-3 ○-2 ○-1 ○0 ○1 ○2 ○3 ○4	( )
	Arousal, A	○-4 ○-3 ○-2 ○-1 ○0 ○1 ○2 ○3 ○4	( )
	Dominance, D	○-4 ○-3 ○-2 ○-1 ○0 ○1 ○2 ○3 ○4	( )
	Satisfaction, S	○-4 ○-3 ○-2 ○-1 ○0 ○1 ○2 ○3 ○4	( )

Schemes No. S-04	Evaluation Indexes	Evaluation Value	Results
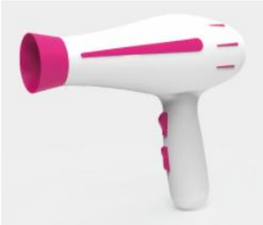	Pleasure, P	○-4 ○-3 ○-2 ○-1 ○0 ○1 ○2 ○3 ○4	( )
	Arousal, A	○-4 ○-3 ○-2 ○-1 ○0 ○1 ○2 ○3 ○4	( )
	Dominance, D	○-4 ○-3 ○-2 ○-1 ○0 ○1 ○2 ○3 ○4	( )
	Satisfaction, S	○-4 ○-3 ○-2 ○-1 ○0 ○1 ○2 ○3 ○4	( )
**Schemes No. S-05**	**Evaluation Indexes**	**Evaluation Value**	**Results**
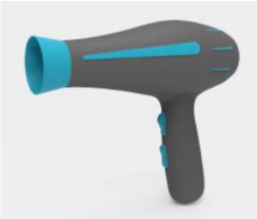	Pleasure, P	○-4 ○-3 ○-2 ○-1 ○0 ○1 ○2 ○3 ○4	( )
	Arousal, A	○-4 ○-3 ○-2 ○-1 ○0 ○1 ○2 ○3 ○4	( )
	Dominance, D	○-4 ○-3 ○-2 ○-1 ○0 ○1 ○2 ○3 ○4	( )
	Satisfaction, S	○-4 ○-3 ○-2 ○-1 ○0 ○1 ○2 ○3 ○4	( )
**Schemes No. S-06**	**Evaluation Indexes**	**Evaluation Value**	**Results**
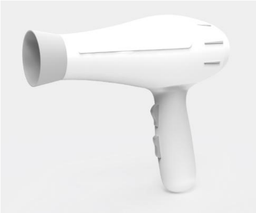	Pleasure, P	○-4 ○-3 ○-2 ○-1 ○0 ○1 ○2 ○3 ○4	( )
	Arousal, A	○-4 ○-3 ○-2 ○-1 ○0 ○1 ○2 ○3 ○4	( )
	Dominance, D	○-4 ○-3 ○-2 ○-1 ○0 ○1 ○2 ○3 ○4	( )
	Satisfaction, S	○-4 ○-3 ○-2 ○-1 ○0 ○1 ○2 ○3 ○4	( )
**Schemes No. S-07**	**Evaluation Indexes**	**Evaluation Value**	**Results**
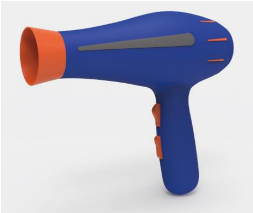	Pleasure, P	○-4 ○-3 ○-2 ○-1 ○0 ○1 ○2 ○3 ○4	( )
	Arousal, A	○-4 ○-3 ○-2 ○-1 ○0 ○1 ○2 ○3 ○4	( )
	Dominance, D	○-4 ○-3 ○-2 ○-1 ○0 ○1 ○2 ○3 ○4	( )
	Satisfaction, S	○-4 ○-3 ○-2 ○-1 ○0 ○1 ○2 ○3 ○4	( )
**Schemes No. S-08**	**Evaluation Indexes**	**Evaluation Value**	**Results**
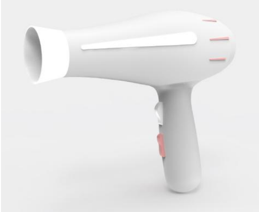	Pleasure, P	○-4 ○-3 ○-2 ○-1 ○0 ○1 ○2 ○3 ○4	( )
	Arousal, A	○-4 ○-3 ○-2 ○-1 ○0 ○1 ○2 ○3 ○4	( )
	Dominance, D	○-4 ○-3 ○-2 ○-1 ○0 ○1 ○2 ○3 ○4	( )
	Satisfaction, S	○-4 ○-3 ○-2 ○-1 ○0 ○1 ○2 ○3 ○4	( )

Schemes No. S-09	Evaluation Indexes	Evaluation Value	Results
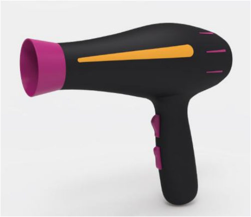	Pleasure, P	○-4 ○-3 ○-2 ○-1 ○0 ○1 ○2 ○3 ○4	( )
	Arousal, A	○-4 ○-3 ○-2 ○-1 ○0 ○1 ○2 ○3 ○4	( )
	Dominance, D	○-4 ○-3 ○-2 ○-1 ○0 ○1 ○2 ○3 ○4	( )
	Satisfaction, S	○-4 ○-3 ○-2 ○-1 ○0 ○1 ○2 ○3 ○4	( )
**Schemes No. S-10**	**Evaluation Indexes**	**Evaluation Value**	**Results**
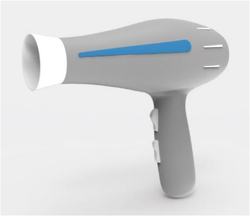	Pleasure, P	○-4 ○-3 ○-2 ○-1 ○0 ○1 ○2 ○3 ○4	( )
	Arousal, A	○-4 ○-3 ○-2 ○-1 ○0 ○1 ○2 ○3 ○4	( )
	Dominance, D	○-4 ○-3 ○-2 ○-1 ○0 ○1 ○2 ○3 ○4	( )
	Satisfaction, S	○-4 ○-3 ○-2 ○-1 ○0 ○1 ○2 ○3 ○4	( )
