# Supplementary material for: A hybrid color emotional experience approach: Integrating the pleasure-arousal-dominance model with fuzzy grey relational analysis
Source: PLoS One. 2026 Feb 2;21(2):e0341895. doi: 10.1371/journal.pone.0341895 (PMC12863556; doi:10.1371/journal.pone.0341895)
Supplement: S3 File — (PDF) [file pone.0341895.s003.pdf]

Fixation Duration Statistics for Color Schemes

Subject No.	Fixation Duration (s)									
	S-01	S-02	S-03	S-04	S-05	S-06	S-07	S-08	S-09	S-10
No.1	10.47	2.84	4.99	5.63	11.56	7.11	4.01	1.49	3.29	6.39
No.2	9.99	1.82	2.51	8.13	7.03	5.87	3.94	5.82	6.96	5.17
No.3	7.39	3.34	5.33	9.99	8.91	4.44	10.84	3.05	1.88	2.09
No.4	5.19	1.89	10.82	4.74	5.51	6.54	8.24	4.01	2.48	4.56
No.5	4.23	2.33	1.91	7.39	8.58	16.43	3.49	6.45	7.16	1.82
No.6	3.92	5.03	4.57	10.69	4.09	7.56	5.69	3.83	5.46	4.47
No.7	7.35	3.33	2.87	3.58	11.24	10.87	9.31	7.27	0.49	3.18
No.8	8.7	5.35	5.89	8.39	5.17	5.44	3.76	3.18	2.77	6.53
No.9	5.97	4.89	3.98	9.18	6.12	10.16	8.30	4.23	1.21	2.82
No.10	3.15	1.61	3.28	7.48	13.9	14.28	4.45	2.29	3.11	3.16
No.11	4.98	3.53	6.13	5.79	4.46	4.93	8.76	3.33	5.35	6.39
No.12	7.74	3.2	3.23	10.75	7.19	11.64	4.32	4.08	2.85	0.39
No.13	11.79	3.46	4.54	4.13	6.41	5.78	6.15	7.03	3.59	3.52
No.14	4.41	0.97	4.11	6.01	15.09	8.61	4.38	1.70	2.92	5.87
No.15	11.78	6.35	3.70	8.76	5.37	6.85	3.95	3.91	2.1	3.16
No.16	4.70	3.34	3.02	11.1	4.61	15.24	4.07	3.18	4.25	0.48
No.17	4.74	2.70	1.02	8.35	12.68	8.78	3.89	4.03	2.20	7.85
No.18	9.52	0.96	4.13	5.12	10.86	5.69	5.04	11.64	2.79	2.97

No.19	4.51	5.91	3.19	9.82	5.42	9.33	4.50	4.30	4.22	3.45
No.20	5.76	2.77	7.46	8.61	6.52	10.22	2.44	3.57	3.96	5.21
No.21	5.11	8.28	2.78	6.24	5.44	16.25	4.72	5.17	1.66	2.61
No.22	3.65	2.45	4.46	5.76	9.26	10.15	9.13	4.42	4.46	4.05
No.23	4.61	5.65	8.91	4.97	5.56	6.49	5.89	2.84	2.34	8.69
No.24	5.29	1.79	5.21	11.89	7.06	10.88	3.79	4.86	3.15	3.12
